# Metatranscriptomic analyses reveal ruminal pH regulates fiber degradation and fermentation by shifting the microbial community and gene expression of carbohydrate-active enzymes

**DOI:** 10.1186/s42523-021-00092-6

**Published:** 2021-04-23

**Authors:** Meng M. Li, Robin R. White, Le Luo Guan, Laura Harthan, Mark D. Hanigan

**Affiliations:** 1 Deptartment of Dairy Science, Virginia Polytechnic Institute and State University, Litton-Reaves Hall, 175 West Campus Drive, Blacksburg, VA 24061 USA; 2 State Key Laboratory of Animal Nutrition, College of Animal Science and Technology, China Agricultural University, Beijing, 100193 P. R. China; 3 Deptartment of Animal and Poultry Science, Virginia Polytechnic Institute and State University, Litton-Reaves Hall, 175 West Campus Drive, Blacksburg, VA 24061 USA; 4 Department of Agricultural, Food and Nutritional Science, University of Alberta, Edmonton, Alberta T6G 2P5 Canada

**Keywords:** Metatranscriptomics, Microbiome, pH, Rumen

## Abstract

**Background:**

Volatile fatty acids (VFA) generated from ruminal fermentation by microorganisms provide up to 75% of total metabolizable energy in ruminants. Ruminal pH is an important factor affecting the profile and production of VFA by shifting the microbial community. However, how ruminal pH affects the microbial community and its relationship with expression of genes encoding carbohydrate-active enzyme (CAZyme) for fiber degradation and fermentation are not well investigated. To fill in this knowledge gap, six cannulated Holstein heifers were subjected to a continuous 10-day intraruminal infusion of distilled water or a dilute blend of hydrochloric and phosphoric acids to achieve a pH reduction of 0.5 units in a cross-over design. RNA-seq based transcriptome profiling was performed using total RNA extracted from ruminal liquid and solid fractions collected on day 9 of each period, respectively.

**Results:**

Metatranscriptomic analyses identified 19 bacterial phyla with 156 genera, 3 archaeal genera, 11 protozoal genera, and 97 CAZyme transcripts in sampled ruminal contents. Within these, 4 bacteria phyla (*Proteobacteria*, *Firmicutes*, *Bacteroidetes*, and *Spirochaetes*), 2 archaeal genera (*Candidatus methanomethylophilus* and *Methanobrevibacter*), and 5 protozoal genera (*Entodinium*, *Polyplastron*, *Isotricha*, *Eudiplodinium*, and *Eremoplastron*) were considered as the core active microbes, and genes encoding for cellulase, endo-1,4-beta- xylanase, amylase, and alpha-N-arabinofuranosidase were the most abundant CAZyme transcripts distributed in the rumen. Rumen microbiota is not equally distributed throughout the liquid and solid phases of rumen contents, and ruminal pH significantly affect microbial ecosystem, especially for the liquid fraction. In total, 21 bacterial genera, 4 protozoal genera, and 6 genes encoding CAZyme were regulated by ruminal pH. Metabolic pathways participated in glycolysis, pyruvate fermentation to acetate, lactate, and propanoate were downregulated by low pH in the liquid fraction.

**Conclusions:**

The ruminal microbiome changed the expression of transcripts for biochemical pathways of fiber degradation and VFA production in response to reduced pH, and at least a portion of the shifts in transcripts was associated with altered microbial community structure.

**Supplementary Information:**

The online version contains supplementary material available at 10.1186/s42523-021-00092-6.

## Background

In the modern cattle industry, high-concentrate diets (50 to 90% grain) are often fed to maintain high milk or meat production. High-concentrate diets can stimulate rumen fermentation by resident microorganisms, producing more volatile fatty acids (VFA) including acetate, propionate, and butyrate and sometimes lactic acid [1, 2]. When VFA or lactic acid accumulate in the rumen, ruminal pH will rapidly drop which is associated with altered microbial ecology and metabolic disorders such as clinical or subclinical rumen acidosis [3–6]. Studies have indicated that the activity or numbers of cellulolytic microbes are inhibited if ruminal pH is less than 6.0, primarily due to the regulation of intracellular pH, resulting in inhibition of cellobiose transport activity [7, 8]. Consequently, fiber degradation and VFA production decrease when pH drops below critical values [9].

Intraruminal VFA production is of paramount importance as it provides up to 75% of total metabolizable energy in ruminants [1, 2], and individual VFA have distinct metabolic fates [9]. The production of VFA shares glycolysis as a common pathway with pyruvate as the central branching point, and conversion of pyruvate to individual VFA is driven by carbohydrate-active enzyme (CAZyme) produced by microorganisms in the rumen [10]. Various in vitro studies have indicated that pH significantly affects the profile and production of VFA [11–13]. Changes in VFA profile and production appear to occur through a shift in the biochemical pathways expressed by the overall microbial population in the rumen. However, the gene expression of CAZyme in response to pH reductions and associations between the rumen microbiota and CAZyme gene expression have not been well investigated.

High-throughput sequencing techniques such as metatranscriptomics can analyze transcripts expressed by a microbial community at a specific point in time, which allows a simultaneous investigation of gene expression and abundance of active microbiomes in an ecosystem [14]. In this work, we used metatranscriptomic analyses to investigate how reduced ruminal pH altered the microbial community, expression of CAZyme transcripts, fiber degradation and VFA concentrations following a continuous 10-day intraruminal acid infusion. We hypothesized that low pH would alter biochemical pathways to affect fiber degradation and VFA production via a shift in microbial community structure and their expressions of CAZyme genes in the rumen.

## Methods

### Animals, experimental design, and feeding management

Six cannulated Holstein heifers with an initial BW of 362 ± 22 kg (mean ± SD) were subjected to each of 2 treatments in a two-period, cross-over design. The treatments were 10 days of continuous intraruminal infusions of distilled water (Control) or a dilute blend of hydrochloric and phosphoric acids to achieve 0.5 unit reduction in pH (LpH). There was a 5-day recovery period between the infusion periods.

The animals were housed in individual tie stalls during the infusions. They had continuous access to water, and were fed a common total mixed ration (TMR) formulated according to National Research Council recommendations [15]. Ingredient composition and nutrient content of the diet are listed in Table S1. The ration was fed every 4 h with approximately 17% of the total daily feed allocated at each feeding to maintain stable rumen fermentation rates. Feed offered and refused was recorded at each feeding time and used to calculate daily feed intake.

The acid solution consisted of 73 g H_3_PO_4_, 185 g HCl and 800 g distilled water. Infusates were delivered into the rumen using an indwelling infusion apparatus and clinical infusion pumps (LifeCare 5000, Abbott Laboratories, North Chicago, IL). Ruminal pH was monitored every 4 h, and the acid infusion rate was varied by animal to achieve a ruminal pH between 6.0 and 6.1. When ruminal pH dropped below 6.0, the infusion rate was decreased by 10 ml/h, and the ruminal pH was rechecked in 30 to 60 min. The infusion rate was adjusted upwards if ruminal pH was above 6.1. Water was infused at a constant rate of 25 ml/h in the Control animals.

### Sample collection

Rumen sampling was conducted by placing 2 small tubes with an 8 mm diameter via the cannula into the rumen contents in different locations within the rumen (cranial and caudal areas of the rumen) to collect rumen fluid prior to each feeding. Four layers of nylon net (approximate 2 mm pore size) were tied around the end of the tubes to filter out the solid fractions. A total of 10 ml of rumen fluid was drawn from the tubes at each sampling event. Ruminal pH was immediately measured using a portable pH meter (Starter 300, Ohaus, Parsippany, NJ), and ruminal liquid samples were stored at − 20 °C until future VFA analyses.

Prior to the morning feeding on day 9 of each period, rumen contents including both liquid and solids were collected via the ruminal cannula for RNA extraction. Ruminal liquid samples were drawn from the tubes. Rumen solid samples were collected from multiple rumen locations (dorsal, ventral, cranial, and caudal areas of the rumen) and excess liquid removed by squeezing through four layers of cheesecloth. The collected liquid and solid samples were immediately flash-frozen in liquid nitrogen, crushed into small pellets, transferred to cryovials, and transported to the laboratory in liquid nitrogen. RNA extraction was completed within 12 h to avoid RNA degradation.

The mixed ration was sampled daily and dried at 55 °C in a forced-air oven for DM determination. Subsamples were ground to pass a 2-mm screen in a Wiley Mill (A.H. Thomas, Philadelphia, PA), and composited by period. A subsample of the 2 mm material was used for further in-situ tests, and an additional subsample was ground through a 1 mm screen (Cyclone lab sample mill, UDY Corporation, Fort Collins, CO) and used for chemical analyses.

### In-situ degradability

In-situ degradation of dietary hemicellulose, cellulose, and lignin were determined using the nylon bag technique described by Ørskov and McDonald [16] on the last 3 days of each period. Briefly, approximately 5 g of dried, ground (2 mm) diet sample was weighed into duplicate 5 × 10 cm polyester bags (50 μm pore size, Ankom Technology, Macedon, NY) and suspended in the rumen in a large (36 × 42 cm) nylon mesh bag secured to the ruminal cannula via a nylon cord. The samples were inserted into the rumen of each heifer before the morning meal and removed after 2, 8, 12, 24, 36, and 48 h of incubation. Degradation rates were estimated as described by Ørskov and McDonald [16] using a non-linear least squares regression procedure (NLI) in R (version 3.5.1) [17]. The equations fitted to the data were:

1 $$ \mathrm{Degraded}(t)=a+b\ \left(1-{e}^{-{K}_dt}\right) $$

2 $$ \mathrm{Effective}\ \mathrm{degradability}\left(\%\right)=\mathrm{a}+\frac{bK_d}{K_d+{K}_p} $$

where *a* represented the soluble fraction (%), *b* represented the potentially degradable fraction (%), *K*_*d*_ represented the degradation rate constant for the b fraction (%/h), *t* represented incubation time in the rumen (h), and *K*_*p*_ was the outflow rate, which was assumed to be 4%/h according to Mertens [18].

### Chemical analyses

Dry matter content was determined according to the National Forage Testing Association method 2.1.4 [19]. Neutral detergent fiber (NDF) was determined as described by Van Soest et al. [20] using heat-stable α-amylase (FAA, Ankom Technology, Macedon, NY) and sodium sulfite. Acid detergent fiber (ADF) and lignin concentrations were determined according to AOAC method 973.18 [21]. Ash content was determined according to AOAC method 942.05 [21]. Hemicellulose was calculated as the difference between NDF and ADF. Cellulose was calculated by subtracting ash and lignin from ADF.

For measurement of ruminal VFA concentrations, rumen fluid samples were thawed and composited by day, animal, and period (*n* = 6). The samples were centrifuged for 30 min at 2500×*g* at room temperature to remove solid particles, and supernatant liquid was collected. An external tracer consisting of a mix of ^13^C-labelled acetate, propionate, and butyrate was added to each liquid sample, then rumen liquid samples were derivatized, and the derivatives were analyzed for isotopic ratio using a Thermo Electron Polaris Q mass spectrometer in tandem with a Thermo Electron Focus gas chromatography (GC-MS; Thermo Electron Corporation, Austin, TX) as described by Kristensen [22].

### RNA extraction and sequencing

RNA from ruminal liquid and solid samples was extracted using an RNA Clean & Concentrator kit from Zymo Research (Irvine, CA, USA), which included a bead-beating step to mechanically break microbial cell walls. DNA was removed by treatment with Baseline-ZERO™ DNase (Epicentre Biotechnologies, Madison, WI) following the manufacturer’s instructions. The removal of DNA was verified by PCR with primers targeting the 16S and 18S rRNA genes. The quality of total RNA was checked using the Agilent 2100 Bioanalyzer (Agilent Technologies, Palo Alto, California). RNA samples with the RNA integrity number (RIN) greater than 7.0 were used for downstream analysis. Concentrations of total RNA were determined using the Qubit® RNA Assay Kit (Thermo Fisher Scientific, Waltham, MA). DNA free, RNA samples were used for library preparation using the TruSeq™ RNA LT Sample Preparation Kit (Illumina). Following library preparation, the final concentration of cDNA in each library was measured using the Qubit® dsDNA HS Assay Kit (Thermo Fisher Scientific, Waltham, MA), and the average library size was determined using the Agilent 2100 Bioanalyzer (Agilent Technologies, Palo Alto, California). The libraries were then pooled in equimolar ratios of 2 nM, and 4 pM of the library pool was clustered using the Illumina’s cBot (Illumina, San Diego, USA). The 150 bp, paired-end sequencing reaction was performed on a HiSeq 2500 platform (Illumina, San Diego, USA) at Molecular Research LP (MRDNA, Shallowater, Texas).

### Transcriptome mapping

The quality of raw paired-end reads was evaluated using the FastQC program (http://www.bioinformatics.babraham.ac.uk/projects/fastqc/). Residual adaptor sequences, low quality bases with quality scores below 20, and reads shorter than 50 bp were removed using the Trimmomatic program (version 0.36) [23]. The 16S and 18S rRNA reads were subsequently extracted from the filtered RNA dataset for taxonomic profiling using the SortMeRNA program (version 2.1) [24] through alignment with the rRNA reference databases SILVA_SSU, SILVA_LSU [25], and the non-coding RNA reference database Rfam 11.0 [26] following descriptions in Li et al. [27]. The remaining filtered reads were aligned to the UMD3.1 *Bos Taurus* reference genome [28, 29] with TopHat2 using the default setting to remove host reads (version 2.1.1) [30]. The filtered reads not matching the host genome were considered putative microbial mRNA and were selected for further functional analyses.

### Taxonomic profiling of the rumen microbial community

The pipeline DADA2 (version 1.6) was used to infer amplicon sequence variants (ASVs) from the aligned total rRNA using R (version 3.4.3;) [17] as described by Callahan et al. [31]. Briefly, the following was completed in sequence. Forward and reverse reads were trimmed with a maximum number of expected errors of 2 based on their quality scores. Error rates were learned using 1 million training sequences each for forward and reverse reads, and the resulting specific error rates for each possible transition (such as A to C, A to G) were used to infer ASVs for each sample from the trimmed reads. The forward and reverse sequences were merged, chimeras were removed, and taxonomy was assigned by comparison to the SILVA database (version 138) [25] using the naïve Bayesian classifier algorithm [32]. The richness of taxa was presented as relative abundance using the phyloseq package (version 1.24.2) [33].

### Identification of genes encoding CAZyme and functional metabolic pathway analysis

The putative microbial mRNA sequences were assembled using Velvet with a kmer size of 31 [34] and aligned with the CAZyme database [35] to annotate glycoside hydrolases (GH) [36–40], glycosyltransferases (GT) [41, 42], carbohydrate binding modules (CBM) [43], polysaccharide lyases (PL), and carbohydrate esterases (CE) [44]. Only the best alignments with expectation values lower than 1× 10^− 4^ were considered for functional gene annotation using the UBLAST algorithm implemented in USEARCH (version 9.2.64) [45]. To remove biases associated with the length of the transcript and the sequencing depth of a sample, transcripts per million (TPM) were used to normalize read count values. The metabolic pathway abundances of each sample were determined from the processed reads using the Human Microbiome Project Unified Metabolic Analysis Network (HUMAnN2) pipeline with default parameters [46]. HUMAnN2 utilized the UniRef and MetaCyc databases to characterize the microbial pathways present in samples, and relative abundances of functional pathways were used for further statistical analysis.

### Statistical analysis

All statistical analyses were conducted using R software (version 3.5.1) [17]. Dry matter intake (DMI), ruminal pH, and VFA concentration data were summarized by day. Ruminal fiber (hemicellulose, cellulose, and lignin) degradability data were summarized by hour within a sampling period. Analysis of variance was conducted in a mixed model with treatment and period as fixed effects and animal as a random effect. Day within animal was included as a repeated measure for DMI, ruminal pH, and VFA concentrations, and hour within animal was included as a repeated measure for ruminal fiber degradability. The interaction between treatment and day or hour was included as a fixed effect in the mixed model. Autoregressive covariance and heterogeneous variance were used in the repeated measures using the nlme package (version 3.1–137) [47]. The kinetic parameters associated with fiber degradation were analyzed using the lmer function in the lme4 package (version 1.1–17) [48].

In the current study, only microbial taxa with a relative abundance greater than 0.05% in at least 25% of populations were considered as being observed and used for the analysis. Because high-throughput sequencing generates compositional data (transcript proportions of total reads rather than absolute values) [49], they do not map to Euclidean space, which can be problematic for statistical analyses [49–52]. Therefore, compositional data need to be transformed before statistical analyses to avoid invalid conclusions [49, 53, 54]. The relative abundance and gene expression data were transformed to a centered log ratio:

3 $$ \left[{x}_1\kern0.5em {x}_2\kern0.5em \dots \kern0.5em {x}_n\right]\Rightarrow \left[\log \left(\frac{x_1}{{\left({x}_1\times {x}_2\dots {x}_n\right)}^{\frac{1}{n}}}+e-1\right)\kern0.5em \log \left(\frac{x_2}{{\left({x}_1\times {x}_2\dots {x}_n\right)}^{\frac{1}{n}}}+e-1\right)\kern0.5em \dots \kern0.5em \log \left(\frac{x_n}{{\left({x}_1\times {x}_2\dots {x}_n\right)}^{\frac{1}{n}}}+e-1\right)\right] $$

where (x_1_ × x_2_ … x_n_)^1/n^ represents the geometric mean of the vector. Zero value components should be excluded when dealing with log ratio transformation. However, the sequencing read counts contained an excessive number of zeros, which presents an obstacle for log ratio transformation [49], results in non-normality [53], can cause spurious correlations [54], and may contribute to high false positive issues [55]. This was resolved by replacement of zero count values prior to transformation based on posterior distribution using a Markov Chain Monte Carlo iterative algorithm in the zCompositions package (version 1.1.1) [56]. We added a constant of e-1 to all ratios prior to transformation to avoid negative values where e is Euler’s number. With such an addition, if the gene or transcript counts equal the geometric mean, the log transformed value equals 1, so log transformed values above 1 indicate read counts greater than the geometric mean, and less than 1 indicate read counts lower than the geometric mean. Thus, the relationships among the features in the taxa and gene expression data were captured in the log ratio abundances, which have the mathematical property of real random variables and can be analyzed using standard statistical methods [49]. Ruminal pH effects within the liquid or solid fraction, and sample fraction effects across the entire pH range were analyzed using orthogonal contrasts in multiple comparisons. Significant differences were declared at *P* < 0.05.

Principal component analysis was performed using the factoextra package [57]. Pairwise correlations were conducted to explore associations between microbes and transcripts encoding CAZyme using the Hmisc package [58]. To decrease the correlation matrix size, rows and columns were filtered if they did not contain at least one correlation coefficient with an absolute value greater than 0.5 and a *P* value less than 0.05. The correlation results were visualized using corrplot package [59] in R.

## Results

### Changes in ruminal community diversity

In total, 417.5 million sequences deriving from 24 samples with an average read length of 155 bp were obtained, with a mean of 17.4 million reads per sample. After removing low quality sequences, 93.7% of reads remained for further processing. After aligning with the rRNA reference databases, 32.6% of sequences were classified as 16S and 18S rRNA, the rest were considered as putative mRNA.

**Fig. 1:**
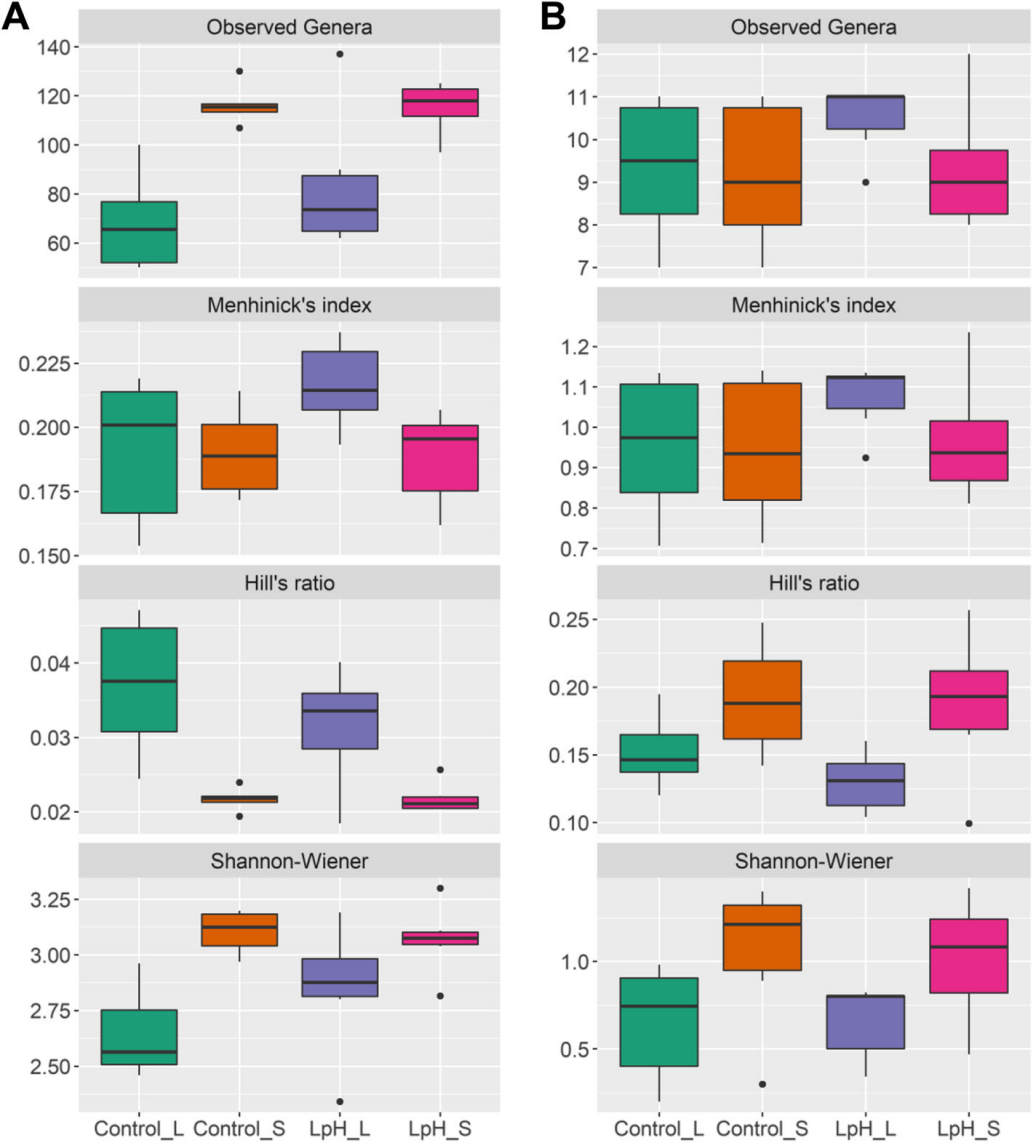
Effects of sampling phases and ruminal pH on observed genera, index of richness, evenness, and alpha diversity among treatments for bacterial (**a**) and protozoal (**b**) genera. Control_L and Control_S represent ruminal liquid and solid fraction at the Control group, and LpH_L and LpH_S represent ruminal liquid and solid fraction in the acid infusion group with a pH reduction of 0.5 units

Approximately 76 and 116 bacterial genera were identified in the ruminal liquid and solid fractions (Fig. 1a and Table S2; *P* < 0.001). A lower pH environment tended to increase numbers of bacterial genera in the liquid fraction compared to normal pH (68 versus 83 bacteria genera; *P* = 0.1), while there was no difference in the solid fraction (116 versus 115 bacteria genera; *P* = 0.87). When considering the total number of individual bacterial taxa, Menhinick’s index indicated low pH increased bacterial richness in the liquid fraction (*P* = 0.02) but did not affect the richness in the solid fraction (*P* = 0.97) compared to the corresponding Control group. Hill’s ratio indicated the there was no pH effect in either fraction (*P* > 0.05). The Shannon-Wiener index indicated that low pH increased bacterial community diversity in the solid fraction compared to the liquid fraction at normal pH (*P* < 0.001), but it had no effect on the solid fraction (*P* = 0.74). Although the liquid fraction had a greater richness than the solid fraction (*P* = 0.04), the liquid fraction was less homogenous than the solid fraction (*P* < 0.001), which contributed to a reduced diversity in the liquid fraction than the solid fraction (*P* < 0.001).

About 10 and 9 protozoal genera were observed in the liquid and solid fractions (Fig. 1b and Table S2). Ruminal pH did not significantly affect richness, evenness, or diversity. Greater evenness and diversity were observed in the solid fraction than the liquid fraction (*P* = 0.004 and 0.005) though the liquid and solid fractions had no difference in richness (*P* = 0.31).

Only 3 archaeal genera were identified in the rumen, and no significant difference was observed in terms of richness, evenness, and diversity between liquid and solid fractions in association with treatment.

### Principle component analysis of bacterial phyla and protozoal genera

**Fig. 2:**
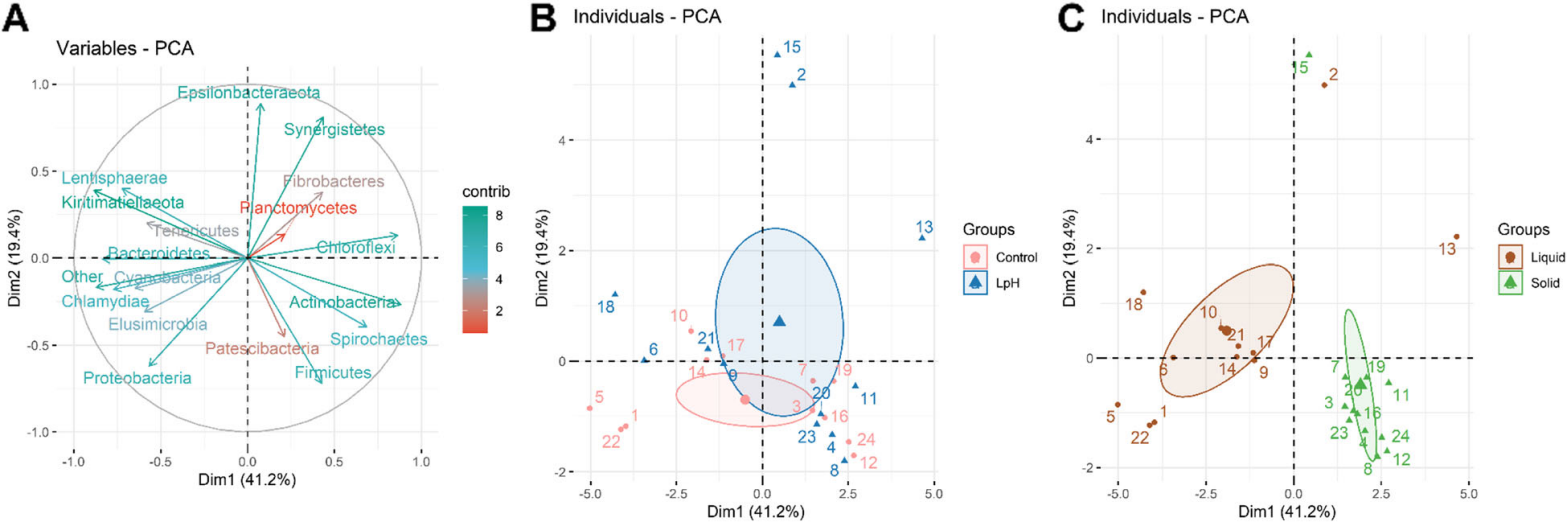
Principal component analyses (PCA) of overall bacterial composition among all samples at the phylum level. Variable contributions to the first two components are labelled with different colors (**a**). All variables were represented by arrows, and individuals were represented by points with numbers. Points were colored by treatment group (**b**) or ruminal sample fraction group (**c**). All the sequence counts were transformed to centered log ratios before PCA analyses

Principle component analysis was conducted to compare overall composition of bacterial phyla among all samples (Fig. 2). The analyses indicated the first component accounted for 41.2% of the total variation, and the second component accounted for 19.4% of the total variation (Fig. 2a). The first component was negatively correlated with *Bacteroidetes* and *Kiritimatiellaeota*, and positively correlated with *Chloroflexi* and *Actinobacteria*; While the second component was negatively correlated with *Firmicutes* and *Proteobacteria*, and positively correlated with *Epsilonbacteraeota*, and *Synergistetes*. Although there was an overlap between the Control and LpH group, the second component can separate different ruminal pH treatments (Fig. 2b), and the first component can clearly separate ruminal liquid and solid fractions (Fig. 2c).

Principle component analysis for protozoal genera was displayed in Figure S1. The first component accounted for 35% of the total variation, and the second component accounted for 24.1% of the total variation. The first component was positively correlated with *Entodinium*, while negatively correlated with *Polyplastron* and *Eudiplodinium* (Figure S1A). There was a positive correlation between the second component and *Dasytricha* and a negative correlation with *Diplodinium*, *Blepharocorys*, and *Cycloposthium* (Figure S1A). Similar to the overall variance structure of bacterial phylum, the second component appeared to separate different ruminal pH treatments (Figure S1B), and the first component separated different ruminal liquid and solid fractions (Figure S1C).

### Changes of taxonomic distribution in the rumen

**Fig. 3:**
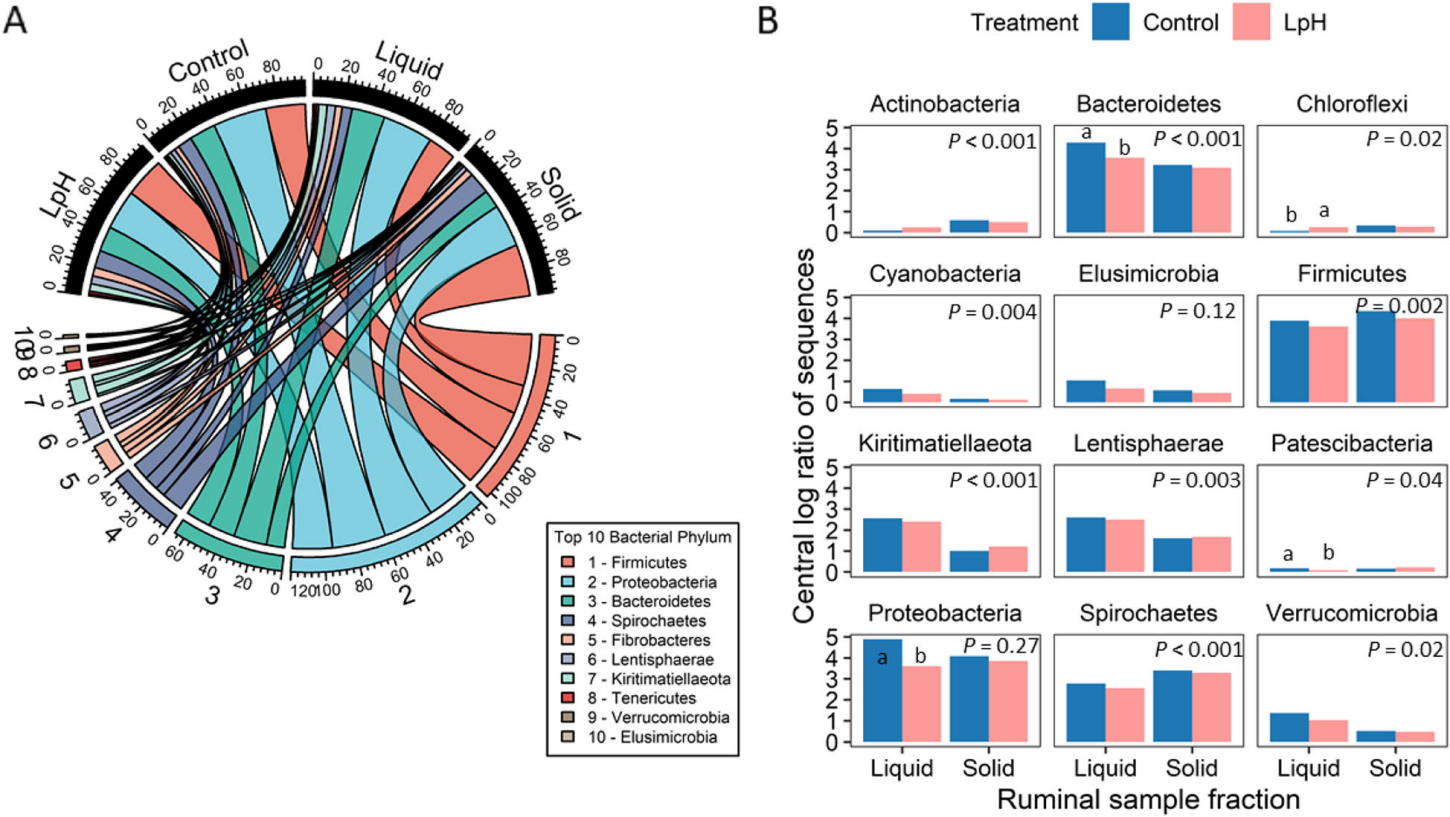
Taxonomic composition of the ruminal microbiome in the liquid and solid fractions in response to normal and low pH treatments. The top 10 bacterial phyla were selected, and sequences were expressed as relative abundance (A). The bar chart shows bacterial phyla that were significantly affected by treatment or by sample fraction (B). *P* value in each block shows difference between the liquid and sloid fraction, and different characters indicate significantly different means within the liquid or solid fraction. Results are reported as central log ratios of sequences where a value of 1 represents the mean ratio

Overall, there were 19 active bacterial phyla with a relative abundance greater than 0.05% identified in all samples. The most abundant phyla were *Firmicutes*, *Proteobacteria*, *Bacteroidetes*, and *Spirochaetes* (Fig. 3A). However, their proportions were dependent on treatments or rumen sample fractions. Approximately 25.5% *Firmicutes*, 34.5% *Proteobacteria*, 17.1% *Bacteroidetes*, and 9.3% *Spirochaetes* were distributed in the liquid fraction of the normal pH group, while there were 26.0% *Firmicutes*, 25.6% *Proteobacteria*, 16.0% *Bacteroidetes*, and 11.1% *Spirochaetes* in the liquid fraction of the LpH group. The population contained 33.2% *Firmicutes*, 27.6% *Proteobacteria*, 11.7% *Bacteroidetes*, and 14.1% *Spirochaetes* in the solid fraction of the Control groups, as compared to 18.2% *Firmicutes*, 32.6% *Proteobacteria*, 21.3% *Bacteroidetes*, and 6.4% *Spirochaetes* in the liquid fraction of the LpH group.

Analysis of variance results were presented in and Fig. 3B and Table S3. The ruminal liquid fraction had greater proportions of *Bacteroidetes*, *Cyanobacteria*, *Kirtimatiellaeota*, *Lentisphaerae*, and *Verrucomicrobia* than that in ruminal solid fractions (*P* < 0.05), but lesser proportions of *Actinobacteria*, *Chloroflexi*, *Firmicutes*, *Patescibacteria*, and *Spirochaetes* (*P* < 0.05). Compared to normal pH, the low pH treatment increased the proportion of *Chloroflexi* in the liquid fraction (*P* = 0.05), and decreased the proportions of *Bacteroidetes*, *Patescibacteria*, and *Proteobacteria* (*P* < 0.05). However, no significant pH effect was observed in the solid fraction.

**Fig. 4:**
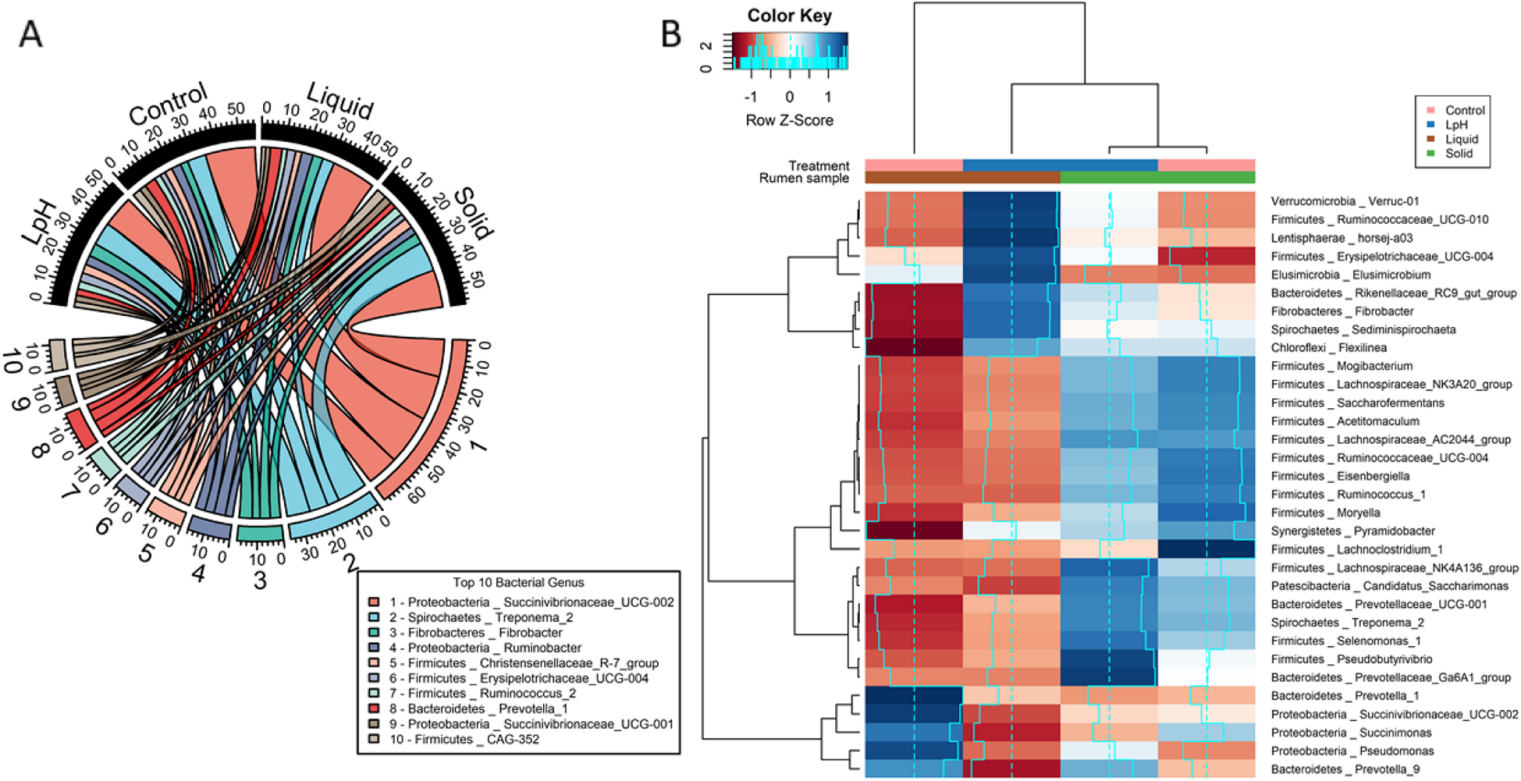
Taxonomic composition of the ruminal microbiome in the liquid and solid fractions in response to normal and low pH treatments. The top 10 bacterial genus were selected, and sequences were expressed as relative abundance (**a**). The heatmap shows bacterial genera that were significantly affected by pH and by sample fraction (**b**). Rows are color coded according to *Z*-score. A *Z*-score change of + 1 is equal to one standard deviation above the row mean. Blue represents upregulation, and red represents downregulation

Analyses at the bacterial genus level were performed to gain further insights into changes in the taxonomic distributions. In total, 156 bacterial genera were identified in the samples, with 61 having relative abundances greater than 0.05% in all the samples. On average, *Succinivibrionaceae_UCG-002*, *Treponema_2*, *Fibrobacter*, *Ruminobacter*, *Christensenellaceae_R-7_group*, *Erysipelotrichaceae_UCG-004*, *Ruminococcus_2*, *Prevotella_1*, *Succinivibrionaceae_UCG-001*, and *CAG-352* were the 10 most abundant genera, accounting for 17.5, 8.9, 4.4, 4.1, 3.6, 3.0, 3.0, 2.9, 2.8, and 2.7% of total bacteria within liquid and solid samples (Fig. 4a). In total, 43 bacterial genera had different proportions between the ruminal liquid and solid samples; 16 bacterial genera were affected by ruminal pH in the liquid fraction; only 5 bacterial genera were affected by pH in the solid fraction (Table S4).

Regardless of ruminal pH, the solid fraction had a greater proportion of *Atopobium*, *Olsenella*, *Prevotellaceae_NK3B31_group*, *Prevotellaceae_UCG-001*, *Prevotellaceae_UCG-004*, *Flexilinea*, *Acetitomaculum*, *Butyrivibrio_2*, *Christensenellaceae_R-7_group*, *Family_XIII_AD3011_group*, *Family_XIII_UCG-001*, *Lachnoclostridium_1*, *Lachnoclostridium_10*, *Lachnospiraceae_AC2044_group*, *Lachnospiraceae_NK3A20_group*, *Lachnospiraceae_NK4A136_group*, *Lachnospiraceae_XPB1014_group*, *Mogibacterium*, *Moryella*, *Pseudobutyrivibrio*, *Ruminococcus_9*, *Ruminococcaceae_UCG-004*, *Ruminococcaceae_UCG-014*, *Ruminococcus_1*, *Ruminococcus_2*, *Saccharofermentans*, *Selenomonas_1*, *Desulfovibrio*, *Treponema_2*, and *Pyramidobacter* than the liquid fraction (*P* < 0.05), and a lesser proportion of *Prevotella_1*, *Prevotellaceae_YAB2003_group*, *Elusimicrobium*, *Anaerovibrio*, *Asteroleplasma*, *Erysipelotrichaceae_UCG-004*, *Bibersteinia*, *Ruminobacter*, *Succinivibrio*, *Sphaerochaeta*, *Synergistes*, *Mycoplasma*, and *Cerasicoccus* (*P* < 0.05).

As displayed in Fig. 4b and Table S4, compared to normal ruminal pH, low ruminal pH decreased *Prevotella*, *Prevotella_9*, *Anaerosporobacter*, *Lachnospiraceae_UCG-007*, *Pseudomonas*, *Succinimonas*, and *Succinivibrionaceae_UCG-002* in the liquid fraction (*P* < 0.05), and increased *Flexilinea*, *Mogibacterium*, *Papillibacter*, *Ruminococcaceae_UCG-010*, *Victivallis*, *Sediminispirochaeta*, *Treponema*, *Pyramidobacter*, and *Synergistes* in the liquid fraction (*P* < 0.05). Finally, low ruminal pH increased *Bifidobacterium* in the solid fraction (*P* < 0.05), and decreased *Lachnoclostridium_1*, *Ruminiclostridium_9*, *Desulfuromonas*, and *M2PT2-76_termite_group* (*P* < 0.05).

In total, 11 protozoal genera were identified through analysis of microbial composition, and 7 of them were observed in all the samples with relative abundances greater than 0.05% of the total population. As displayed in Figure S2, *Entodinium*, *Polyplastron*, *Isotricha*, *Eudiplodinium*, and *Eremoplastron* were highly abundant representing 67.9, 11.0, 9.6, 2.7, and 2.9% of the population in all the liquid and solid samples. The ruminal liquid fraction had lesser proportions of *Diploplastron* and *Eudiplodinium* than the solid fraction (*P* < 0.05; Table S5). Low pH decreased the proportion of *Entodinium* and *Isotricha* in the liquid samples (*P* < 0.05; Table S5). However, no significant pH effect was observed in the solid samples.

*Candidatus methanomethylophilus* and *Methanobrevibacter* were the most abundant archaeal genera, accounting for approximately 54.3 and 25.1% of total ruminal archaea. Low ruminal pH did not change archaeal composition in either fraction. However, the ruminal solid fraction had a greater proportion of *Methanobrevibacter* than the liquid fraction (*P* < 0.05), and a lesser proportion of *Candidatus methanomethylophilus* (*P* < 0.05; Table S6).

### Changes in CAZyme transcripts expressed by rumen microbiota

**Fig. 5:**
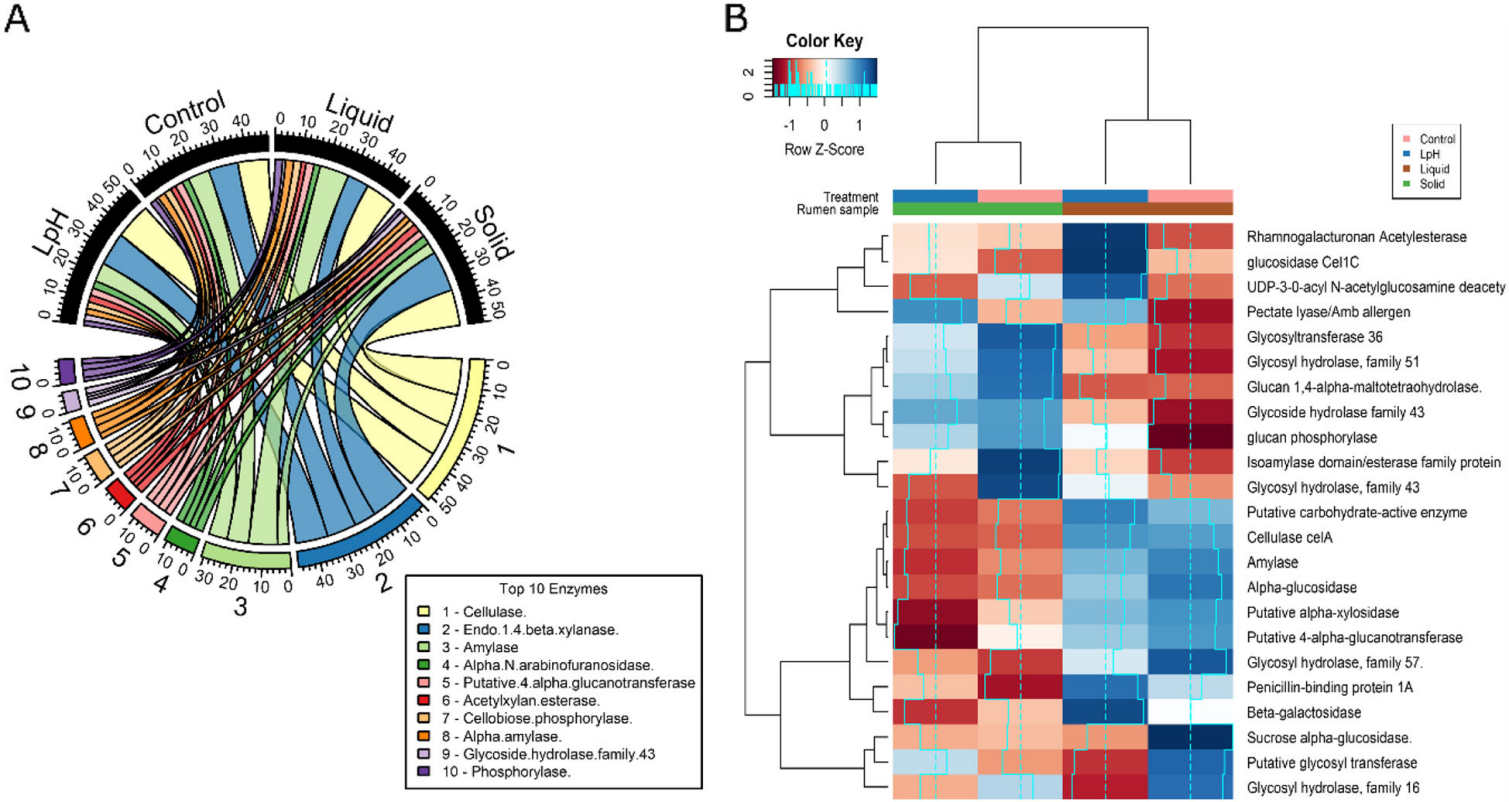
mRNA expressions of the carbohydrate-active degrading enzymes. The top 10 enzymes were selected (**a**). Sequence counts were expressed as relative abundance (%). Heatmap shows enzymes that were significantly affected by pH effect and sample fractions (**b**). Rows are color coded according to Z-score. A Z-score change of + 1 is equal to one standard deviation above the row mean. Blue represents upregulation, and red represents downregulation

In total, 97 transcripts encoding CAZyme were identified, and 88 had a relative abundance above 0.05% in all the samples. As displayed in Fig. 5a, genes encoding cellulase, endo-1,4-beta- xylanase, amylase, and alpha-N-arabinofuranosidase were the most abundant transcripts in the liquid and solid fractions, accounting for 12.83, 11.87, 7.72, and 2.75% of the total enzyme transcripts. As shown in Table S7 and Fig. 5b, 8 transcripts were significantly affected by ruminal pH in the liquid fraction; 2 transcripts were influenced by ruminal pH in the solid fraction, and 16 transcripts had different distributions between the liquid and solid fractions. Within them, 2 transcripts were affected by both pH in the liquid fraction and sample effect, and 1 was affected by ruminal pH in both liquid and solid fractions.

The expression of genes encoding glucan phosphorylase, glucosidase Cel1C, pectate lyase, rhamnogalacturonan acetylesterase, and UDP-3-0-acyl N-acetylglucosamine deacetylase (accession number: ACM90985.1, AAP30745.1, EC 4.2.2.2, CAA61858.1, and ADE83477.1) were upregulated by lower ruminal pH in the liquid fraction (*P* < 0.05), while transcripts of glycosyl hydrolase family 16, putative glycosyl transferase, and sucrose alpha-glucosidase (accession number: ADE81965.1, ADE81144.1, and EC 3.2.1.48) were downregulated (*P* < 0.05). Compared to the normal pH, the low pH environment decreased expression of genes encoding glycosyl hydrolase family 43 (accession number: ADE82026.1) in the solid fraction (*P* < 0.05), while increasing gene expression of pectate lyase (accession number: EC 4.2.2.2; *P* < 0.05). The ruminal liquid fraction contained a greater proportion of transcripts of alpha-glucosidase, amylase, beta-galactosidase, cellulase celA, glycosyl hydrolase family 57, penicillin-binding protein 1A, putative 4-alpha-glucanotransferase, putative alpha-xylosidase, putative carbohydrate-active enzyme, and sucrose alpha-glucosidase (accession number: EC 3.2.1.20, EC 3.2.1.1, EC 3.2.1.23, EC 3.2.1.4, ADE82175.1, ADE81110.1, ADE83753.1, ADE83753.1, ADE82775.1, ADD61402.1, and EC 3.2.1.48) than the solid fraction (*P* < 0.05), while lesser transcripts of glucan 1,4-alpha-maltotetraohydrolase, glucan phosphorylase, glycoside hydrolase family 43, glycosyl hydrolase family 51, glycosyltransferase 36, and isoamylase domain/esterase family protein (accession number: EC 3.2.1.60, ACM90985.1, ACX75355.1, ADE81862.1, ADU20744.1, and ADE82534.1; *P* < 0.05).

### Functional metabolic pathway analysis

In total, 51 metabolic pathways were identified through the HUMAnN2 pathway analysis, and 5 pathways were significantly different among treatments (Table 1). Low pH significantly decreased relative abundances of metabolic pathway participated in glycolysis I (from glucose 6-phosphate), glycolysis II (from fructose 6-phosphate), pyruvate fermentation to acetate and lactate, and pyruvate fermentation to propanoate in the liquid fraction (*P* < 0.05). However, no significant difference was observed in the solid fraction. The relative abundances of metabolic pathway related to glycolysis I (from glucose 6-phosphate), L-isoleucine biosynthesis I (from threonine), and pyruvate fermentation to acetate and lactate were greater in the solid fraction than in the liquid fraction (*P* < 0.05).

**Table 1:** Functional pathways that were significantly different among treatments^a^

Pathway	Control		LpH		SEM	*P* value		
	Liquid	Solid	Liquid	Solid		Con vs LpH^b^	Con vs LpH^c^	Liquid vs Solid^d^
GLYCOLYSIS: glycolysis I (from glucose 6-phosphate)	0.25	0.33	0.09	0.28	0.07	0.05	0.53	0.03
PWY-5484: glycolysis II (from fructose 6-phosphate)	0.23	0.28	0.08	0.22	0.07	0.04	0.43	0.06
ILEUSYN-PWY: L-isoleucine biosynthesis I (from threonine)	0.12	0.27	0.07	0.24	0.05	0.50	0.60	0.002
PWY-5100: pyruvate fermentation to acetate and lactate	0.20	0.25	0.06	0.23	0.06	0.04	0.72	0.03
P108-PWY: pyruvate fermentation to propanoate	0.67	0.56	0.25	0.49	0.12	0.03	0.69	0.60

^a^Relative abundances of functional pathways were transformed to centered log ratio to avoid compositional data problem

^b^Control versus LpH within the ruminal liquid fraction

^c^Control versus LpH within the ruminal solid fraction

^d^Ruminal sample fraction effect regardless of ruminal pH

### Intake, fiber degradation, and VFA concentrations

**Fig. 6:**
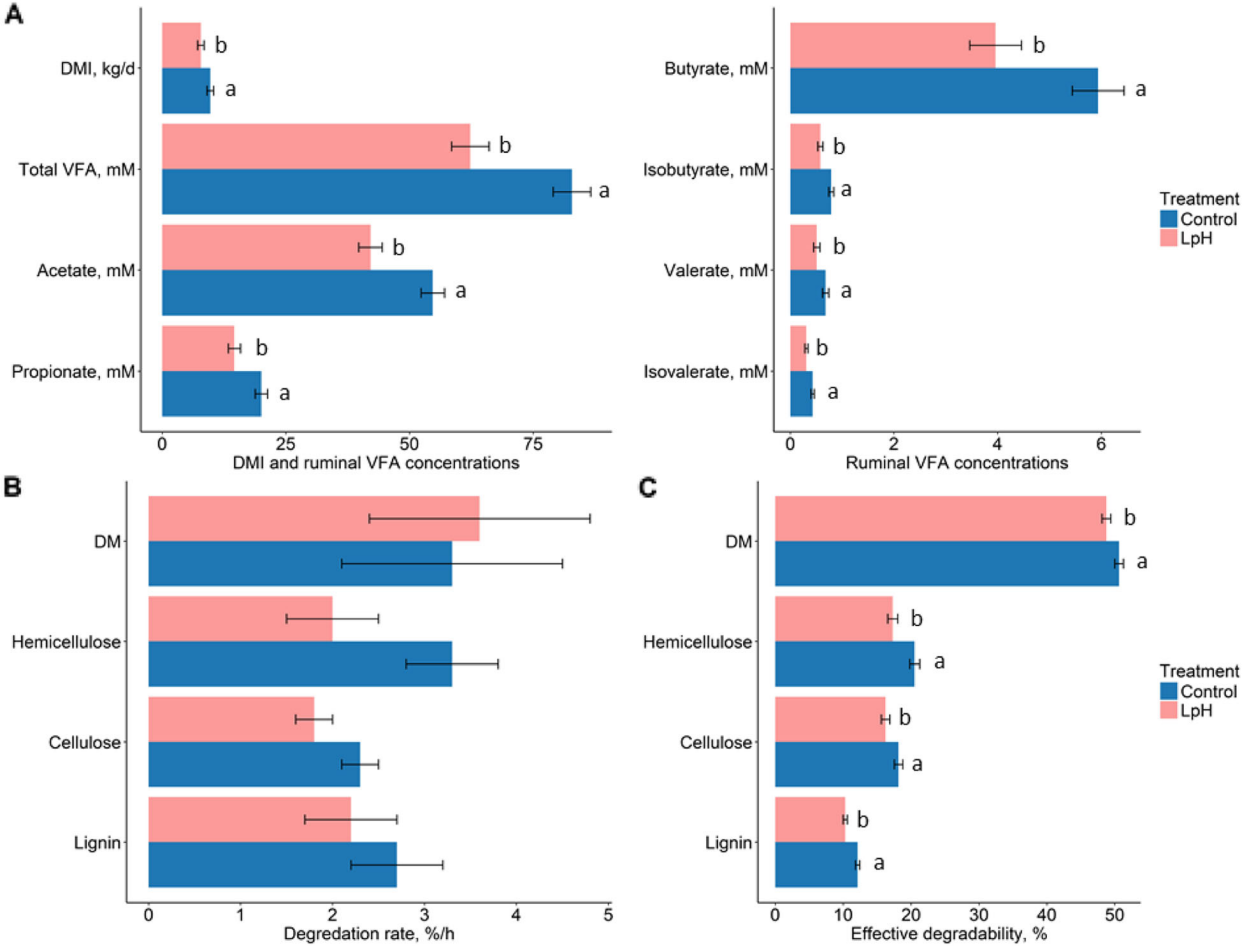
Effects of ruminal pH on DMI, fiber degradation, and ruminal volatile fatty acid concentrations. Different characters indicate significantly different means between normal and low pH treatment

Real time pH over the whole experimental period were displayed in Figure S3. As designed, the mean ruminal pH achieved for the Control and LpH treatments were 6.44 and 6.09, respectively. Compared to the Control, DMI was inhibited by decreasing ruminal pH (*P* = 0.04, Fig. 6A and Table S8).

In situ degradation of dietary DM, hemicellulose, cellulose, and lignin with respect to the rumen incubation time were displayed in Figure S4. Although the individual parameters *a*, *b*, and *k*_*d*_ were not affected by lower ruminal pH (Fig. 6B and Table S8), effective degradabilities of dietary DM, hemicellulose, cellulose, and lignin were decreased (*P* < 0.05, Fig. 6C). Decreased DMI and ruminal fiber degradability were associated with decrease of VFA concentrations and presumably production rates. As a result, concentrations of ruminal total VFA, acetate, propionate, butyrate, isobutyrate, valerate, and isovalerate were decreased in response to lower ruminal pH (*P* < 0.05; Fig. 6A and Table S8).

### Correlations between ruminal microbes and gene expression of CAZyme

**Fig. 7:**
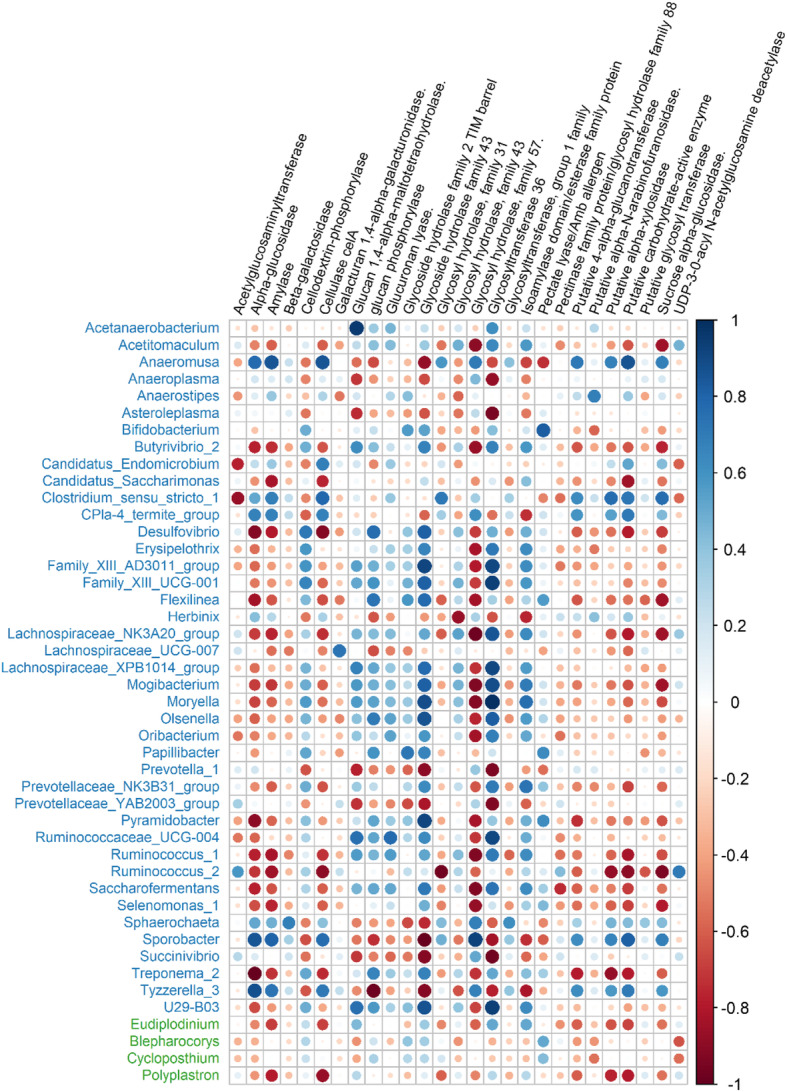
Pairwise correlations between microbial genera and carbohydrate-active degrading enzymes. Only the enzyme expressions that were significantly correlated with bacterial genera were shown (*P* value < 0.05, |r| > 0.5). The bacterial genera were colored by blue, and protozoan genera were colored by green

Pairwise correlations between microbes and transcripts encoding CAZyme were displayed in Fig. 7. There were 45 microbes (41 bacterial genera and 4 protozoal genera) and 27 CAZyme transcripts that had at least one correlation coefficient above 0.5 or less than − 0.5 which was the criteria for inclusion in the matrix regardless of ruminal liquid or solid fractions. However, there were no significant correlations identified among archaeal genera and transcripts encoding CAZyme.

## Discussion

### The distribution of active microbes in the rumen

While metagenomics can reflect the comprehensive diversity of all the active and inactive microorganisms, metatranscriptomics is a more reliable tool to obtain insights into the most active microorganisms. In the present study, *Proteobacteria*, *Firmicutes*, *Bacteroidetes*, and *Spirochaetes* were the most predominant active bacterial phyla regardless of liquid and solid fractions in the rumen. These results were consistent with bacterial phylum profiles in beef cattle under metatranscriptomic analyses [60]. Although the same phyla were identified as dominant bacteria in metagenomic studies, their community structure is quite different with approximately 50.5, 29.8, and 10.6% of *Firmicutes*, *Bacteroidetes*, and *Proteobacteria* [61–64], implying there was a difference of bacterial community structure at the genomic and transcriptomic levels. Kang et al. [65] indicated the abundance of *Proteobacteria* was greater when derived from rumen RNA than from DNA, and this difference was validated using denaturing gradient gel electrophoresis (DGGE) and qRT-PCR techniques. These findings may help explain observations herein of *Proteobacteria* being the dominant active phyla in the rumen.

Besides differences at the genomic and transcriptional levels, bacterial community structure can be influenced by diets or animal breed. Henderson et al. [63] indicated that *Prevotella*, *Butyrivibrio*, and *Ruminococcus*, as well as unclassified *Lachnospiraceae*, *Ruminococcaceae*, *Bacteroidales*, and *Clostridiales* are considered the core microbiome at the genus level across a wide geographical range. Li et al. [66] reported that *Prevotella* (11.94%), *Treponema* (11.25%), unnamed *Succinivibrionaceae* (8.98%), unclassified *Bacteroidales* (6.05%), and *Fibrobacter* (6.01%) were the most abundant active bacterial genera in the rumen. Although most of these genera were also identified as dominant taxa in the current study, their proportions were quite different, suggesting that the bacterial community structure could be caused by diet or breed effect, as a high grain diet was fed to beef cattle in the previous study [66].

As expected, no unique taxonomic groups were identified for the solid and liquid environment, since they are prone to continuous interaction and mutual influences [64]. Substantial differences in terms of the relative abundance of specific taxa were observed. The difference in microbial composition has previously been observed to be associated with substrate availability [67–69], rumen kinetics with respect to particle size [70], and physical and chemical properties [71]. We found that free-floating bacteria that readily degrade metabolizable carbohydrates, such as *Bacteroidetes* and *Lentisphaerae*, were more prevalent in the liquid fraction, while the cellulolytic bacteria, such as *Firmicutes* and *Spirochaetes*, are prominent members in the solid fraction. These results were consistent with previous studies [64, 69, 72].

*Candidatus methanomethylophilus* and *Methanobrevibacter* were the most abundant archaeal genera based on transcript activity. Similar results were reported by Wang et al. [73] for black goats. A greater proportion of the archaeal genus *Methanobrevibacter* was observed in the ruminal solid fraction compared with the liquid fraction, while *Candidatus methanomethylophilus* were more abundant in the liquid than the solid fraction. Similar results were also reported by Henderson et al. [74] and De Mulder et al. [64]. The location differences are related to their potential metabolic functions. Borrel et al. [75] published the genome sequence of *Candidatus methanomethylophilus* isolated from human gut and reported this isolate had genes for methylotrophic methanogenesis from methanol and methylamines. *Methanobrevibacter* is a methanogen related to the bioconversion of cellulose fiber to methane through a symbiotic relationship with a rumen anaerobic fungus [76].

In the current study, *Entodinium* was the predominant protozoa genus in the rumen, which was consistent with previous studies [6, 77, 78], which has been characterized as a starch feeder. Maltase and amylase activity was prevalent in the cell free extracts made from the *Entodinium* suspensions [79]. However, the rest of the protozoal genera were highly varied. Henderson et al. [63] demonstrated the variability of protozoa between and within cohorts of co-located animals was much greater than bacteria and archaea. We found that ruminal liquid had a lower proportion of *Diploplastron* and *Eudiplodinium* than the solid fraction. However, De Menezes et al. [80] observed that protozoan communities were very similar between ruminal liquid and solid fractions. The protozoal community was affected by rumen sample fractions which might be caused by the factors related to ruminal dynamics and protozoal growth. Inversely related to rumen retention time, liquid associated protozoa have a greater ruminal outflow rate than solid associated protozoa, which might result in a greater proportion of protozoa in the solid fraction.

### Microbial community changes in response to ruminal pH

A reduction in ruminal pH by 0.5 units decreased the proportions of *Bacteroidetes*, *Patescibacteria*, and *Proteobacteria* in the liquid fraction but did not affect their proportions in the solid fraction, suggesting that *Bacteroidetes*, *Patescibacteria*, and *Proteobacteria* were less sensitive to low ruminal pH in the solid fraction than the liquid fraction. Schulze et al. [81] demonstrated that VFA concentrations declined and pH increased as the sampling location moved from the medial to the ventral part of the rumen. This was ascribed to greater rates of microbial fermentation occurring in the medial part of the rumen, while VFA absorption by the rumen epithelium occurs in the ventral part of the rumen. This pH gradient may explain the relative lack of a response to pH in the solid fraction. Microbes in that fraction may be selected for tolerance to low pH.

Petri et al. [61] reported that the relative abundance of *Prevotella*, *Acetitomaculum*, *Pseudobutyrivibrio*, *Selenomonas*, *Succinivibrio*, *Treponema*, and *vadinHA42* genera in the rumen fluid increased following a high grain diet challenge. Plaizier et al. [82] found the abundance of *Succinivibrio* in the rumen fluid increased when animals were challenged with a high grain diet. Surprisingly, none of these genera were significantly affected in our study except for *Treponema*, implying different mechanisms of action leading to bacterial community shifts in response to increased grain load versus increased H^+^ concentrations. High grain diets provide a non-structural carbohydrate substrate that results in greater energy for maintenance and growth of microbes with reduced pH occurring as a consequence of that fermentative activity. Conversely, acid infusion reduced ruminal pH without a change in substrate supply. Increased H^+^ concentrations have been found to increase the transmembrane pH gradient and electrical potential, which requires energy to maintain physiological gradient given increased passive transport driven by the electrical gradient [7, 12]. Therefore, the increased maintenance activity might have contributed to inhibited transport activity and reduced microbial growth [7].

Franzolin and Dehority [83] reported that feeding a high concentrate diet increased the proportions of the protozoal genera *Isotricha* and *Epidinium*. Hook et al. [84] found that a high concentrate diet increased the number of *Entodinium*, *Ophryoscolex*, *Isotricha*, and *Dasytricha*. These responses imply that any inhibition in growth caused by reduced pH is overwhelmed by the response to increased substrate supply. This is consistent with our observations of decreased proportions of *Isotricha* and *Entodinium* in the liquid fraction with low ruminal pH in the absence of increased substrate supply.

### The distribution of CAZyme transcripts in the rumen and associations with rumen microbes

Genes encoding for cellulase, endo-1,4-beta-xylanase, amylase, and alpha-N-arabinofuranosidase were the dominant enzyme transcripts in the rumen, which was consistent with previous studies. Cellulase has been assigned to multiple carbohydrate binding module families (CBM), and glycoside hydrolase families (GH), which hydrolyzes 1,4-beta-D-glycosidic linkages to release individual monosaccharides [85]. Wang et al. [86] indicated GH5 and GH9 were the most frequent cellulases found in a metagenomic study. Similar results were also reported in metatranscriptomic studies [10, 87]. Williams et al. [88] indicated that glycosyl hydrolases 5 and 11, polysaccharide lyases and deacetylases, xylanases were the most highly expressed CAZyme transcripts in isolated rumen protozoa, suggesting that protozoa have a significant contribution to carbohydrate breakdown in the rumen. Endo-1,4-beta-xylanase catalyzes the hydrolysis of glycosidic linkages in the xylan backbone [89], and alpha-N-arabinofuranosidase cleaves arabinose from the xylose backbone. The biological functions of cellulase, endo-1,4-beta-xylanase, and alpha-N-arabinofuranosidase are responsible for the degradation of cellulose and hemicellulose in diets, which are consistent with the distribution of cellulolytic rumen bacteria and protozoa in the rumen.

Amylases are a group of enzymes that hydrolyze glycosidic bonds present in starch, which have been grouped into multiple CBM families and GH13, 14, 15, 31, and 57. Deusch et al. [69] observed that GH 57 was the most abundant family across all samples in the rumen. Comtet-Marre et al. [90] reported amylases represent 20% of total GH in the rumen of dairy cows.

Carbohydrate-active enzymes are generally secreted by ruminal microbes. Based on this, the distribution of CAZyme genes would be expected to be associated with the locations of the microbial community. Correlations between rumen microbes and enzyme transcripts indicated that the microbial community patterns were strongly associated with the gene expression patterns in the liquid and solid sampling locations. Thus, our hypothesis of colocalization of microbes possessing genes for specific enzymes and the expression of such enzyme transcripts was supported. Meanwhile, some enzyme transcripts (such as amylase, cellulase celA, and putative alpha-xylosidase) were strongly correlated with both bacterial and protozoal taxa, implying that horizontal gene transfer (HGT) might have been introduced. Ricard et al. [91] demonstrated that the rumen protozoa could acquire many of their CAZymes via HGT, showing significant levels of similarity to the original bacterial genes. Williams et al. [88] also provided evidence of significant contribution that the protozoa make to carbohydrate breakdown in the rumen acquired from the rumen bacteria potentially using HGT.

### Nutritional consequence of low pH regulation

Dietary carbohydrates including hemicellulose, cellulose, and starch, are the primary fermentation substrates in the rumen. They are degraded to hexoses and pentoses, and fermented to VFA via pyruvate [2]. Previous studies indicated VFA concentrations were reliable indexes for the relative production rates [92, 93], although the manipulation of pH independent of VFA production in the current study may have partially delinked production and concentrations due to potential stimulation of transport activity [94]. France and Dijkstra [2] demonstrated that fermentation patterns are determined by the composition of the microbial population which is driven by substrate composition. Regardless of whether fermentation shifts due to a change in microbial structure or due to a change in the expression patterns of a constant structure, characterization of the transcriptome should provide insight into the pathways being used and the microbes expressing those genes.

Low ruminal pH for prolonged periods in the current work negatively affected DMI, fiber degradation, and VFA concentrations, which agreed with previous studies [3, 9, 13, 84, 95]. Stewart [96] reported that reducing pH from 7.0 to 6.0 inhibited cellulolytic activity in the rumen. Hu et al. [97] found an inhibitory effect of low pH on cellulose degradation when pH was below 6.0. Sung et al. [95] showed that lowering incubation media pH to 5.7 decreased bacteria attached to substrate. In the current study, low ruminal pH reduced proportions of metabolic pathway participated in glycolysis, pyruvate fermentation to acetate, lactate, and propanoate in the liquid fraction. At least a portion of the shift in the metabolic pathways was associated with the altered microbial structure. Therefore, low pH could alter metabolic pathways to affect fiber degradation and VFA production via a shift in gene expression expressed by the microbes in the rumen.

## Conclusions

Ruminal pH associated with sampling location of the rumen contents significantly affected the microbial ecosystem. Sixteen bacterial genera and 2 protozoal genera were affected by low ruminal pH in the liquid fraction, However, only 5 bacterial genera and none of protozoal genera were affected by low pH in the solid fraction, implying that microbes exhibited different acid resistance in the liquid and solid fraction. Forty-three bacterial genera, 2 protozoal genera, and 2 archaeal genera exhibited different proportions between ruminal liquid and solid fractions, which suggest that microbiota are not equally distributed throughout the liquid and solid phases of rumen contents.

Low ruminal pH for prolonged periods downregulated CAZyme transcripts and metabolic pathways associated with glycolysis and pyruvate fermentation, leading to decreased fiber degradation and VFA production, suggesting that the ruminal microbiome changed the expression of transcripts in response to reduced pH, and at least a portion of the shifts in transcripts was associated with altered microbial structure.

## Supplementary Information

**Additional file 1: Figure S1.** Principal component analyses (PCA) of overall protozoal composition among all samples at the general level. Variable contributions to the first two components are labelled with different colors (A). All variables were represented by arrows, and individuals were represented by points with numbers. Points were colored by treatment group (B) or ruminal sample fraction group (C). All the sequence counts were transformed to centered log ratios before PCA analyses. **Figure S2.** Taxonomic composition of the ruminal microbiome in the liquid and solid fractions in response to high and low pH. The top 10 protozoal genera were selected. Sequences were expressed as relative abundance. **Figure S3.** Ruminal pH achieved during each of 10-day period. **Figure S4.** In situ degradation of dietary DM, hemicellulose, cellulose, and lignin with respect to the rumen incubation time. **Table S1.** Ingredient composition and nutrient content of the ration^1^. **Table S2.** Effects of sampling site and ruminal pH on index of richness, alpha diversity, and evenness among treatments at bacterial and protozoal genera level. **Table S3.** The effect of sampling site and treatment on bacterial phyla^1^. **Table S4.** Bacterial genera that were significantly different among treatments^1^. **Table S5.** Protozoal genera that differed among treatments^1^. **Table S6.** Archaea genera that differed among treatments^1^. **Table S7.** Carbohydrate-active degrading enzymes that differed among treatments^1^. **Table S8.** Effects of ruminal pH on DMI and ruminal short chain fatty acid concentrations. **Table S9.** Effects of ruminal pH change on in situ fiber degradation kinetics^1^.

## Data Availability

All raw sequence data have been deposited in the NCBI Sequence Read Archive under accession number PRJNA497850.

## Abbreviations

DMI: Dry matter intake
VFA: Volatile fatty acids
TMR: Total mixed ration
CAZyme: Carbohydrate-active enzyme
HGT: Horizontal gene transfer
