# Supplementary material for: Metatranscriptomic analyses reveal ruminal pH regulates fiber degradation and fermentation by shifting the microbial community and gene expression of carbohydrate-active enzymes
Source: Anim Microbiome. 2021 Apr 23;3:32. doi: 10.1186/s42523-021-00092-6 (PMC8063335; doi:10.1186/s42523-021-00092-6)
Supplement: Supplementary file 1 — Additional file 1: Figure S1. Principal component analyses (PCA) of overall protozoal composition among all samples at the general level. Variable contributions to the first two components are labelled with different colors (A). All variables were represented by arrows, and individuals were represented by points with numbers. Points were colored by treatment group (B) or ruminal sample fraction group (C). All the sequence counts were transformed to centered log ratios before PCA analyses. Figure S2. Taxonomic composition of the ruminal microbiome in the liquid and solid fractions in response to high and low pH. The top 10 protozoal genera were selected. Sequences were expressed as relative abundance. Figure S3. Ruminal pH achieved during each of 10-day period. Figure S4. In situ degradation of dietary DM, hemicellulose, cellulose, and lignin with respect to the rumen incubation time. Table S1. Ingredient composition and nutrient content of the ration1. Table S2. Effects of sampling site and ruminal pH on index of richness, alpha diversity, and evenness among treatments at bacterial and protozoal genera level. Table S3. The effect of sampling site and treatment on bacterial phyla1. Table S4. Bacterial genera that were significantly different among treatments1. Table S5. Protozoal genera that differed among treatments1. Table S6. Archaea genera that differed among treatments1. Table S7. Carbohydrate-active degrading enzymes that differed among treatments1. Table S8. Effects of ruminal pH on DMI and ruminal short chain fatty acid concentrations. Table S9. Effects of ruminal pH change on in situ fiber degradation kinetics1. [file 42523_2021_92_MOESM1_ESM.docx]

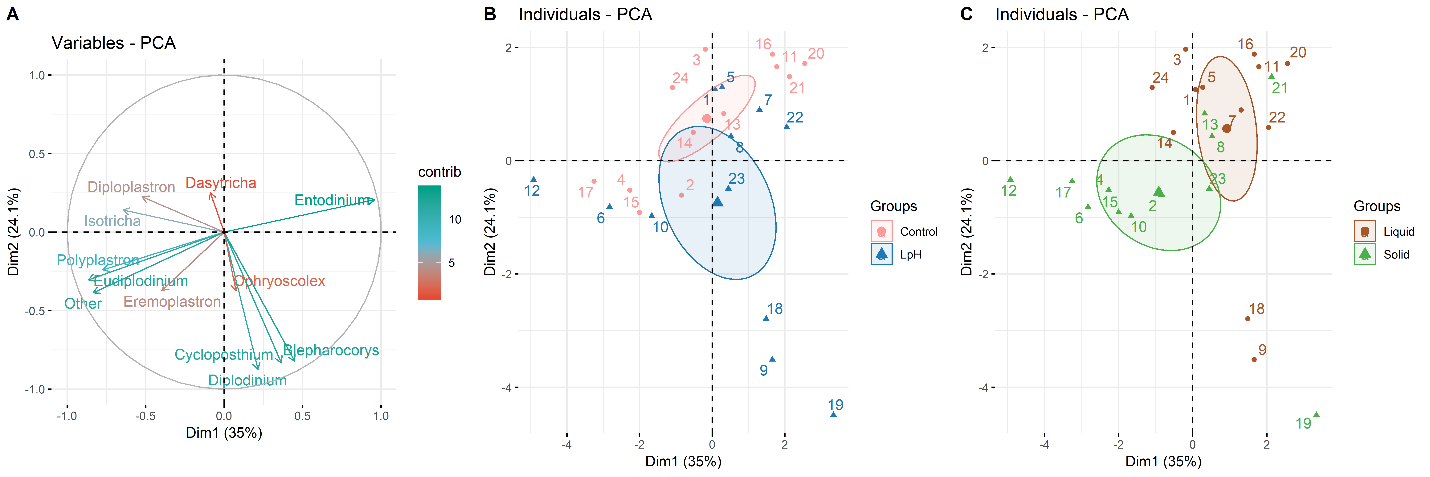


Figure S1. Principal component analyses (PCA) of overall protozoal composition among all samples at the general level. Variable contributions to the first two components are labelled with different colors (A). All variables were represented by arrows, and individuals were represented by points with numbers. Points were colored by treatment group (B) or ruminal sample fraction group (C). All the sequence counts were transformed to centered log ratios before PCA analyses.


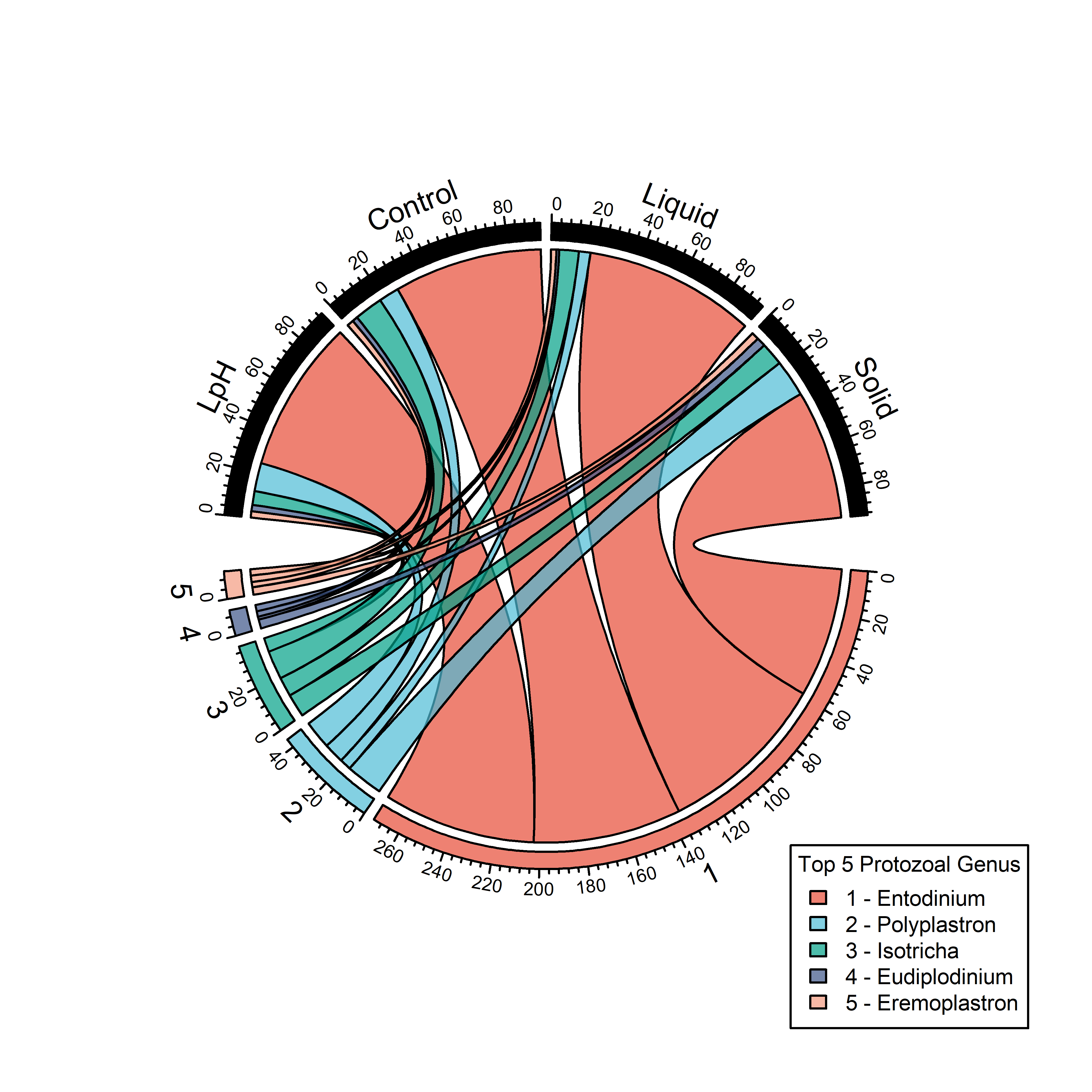


Figure S2. Taxonomic composition of the ruminal microbiome in the liquid and solid fractions in response to high and low pH. The top 10 protozoal genera were selected. Sequences were expressed as relative abundance.


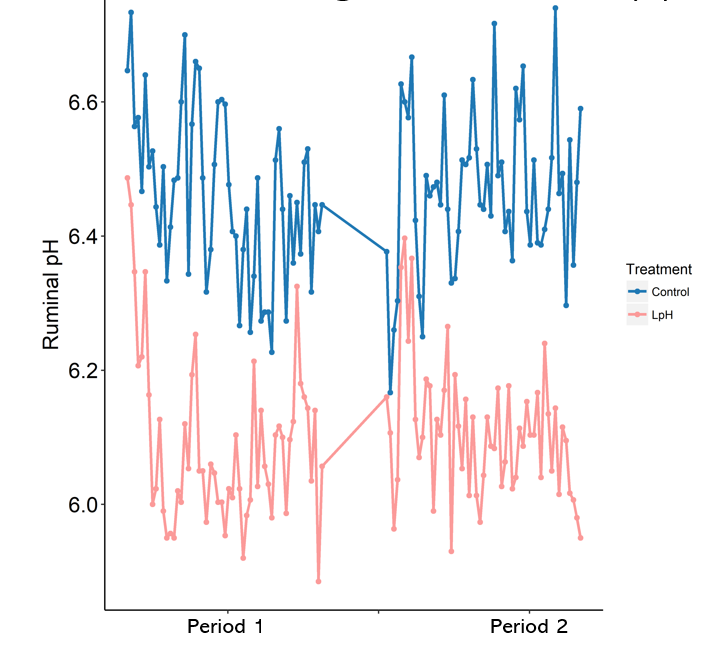


Figure S3. Ruminal pH achieved during each of 10-day period.


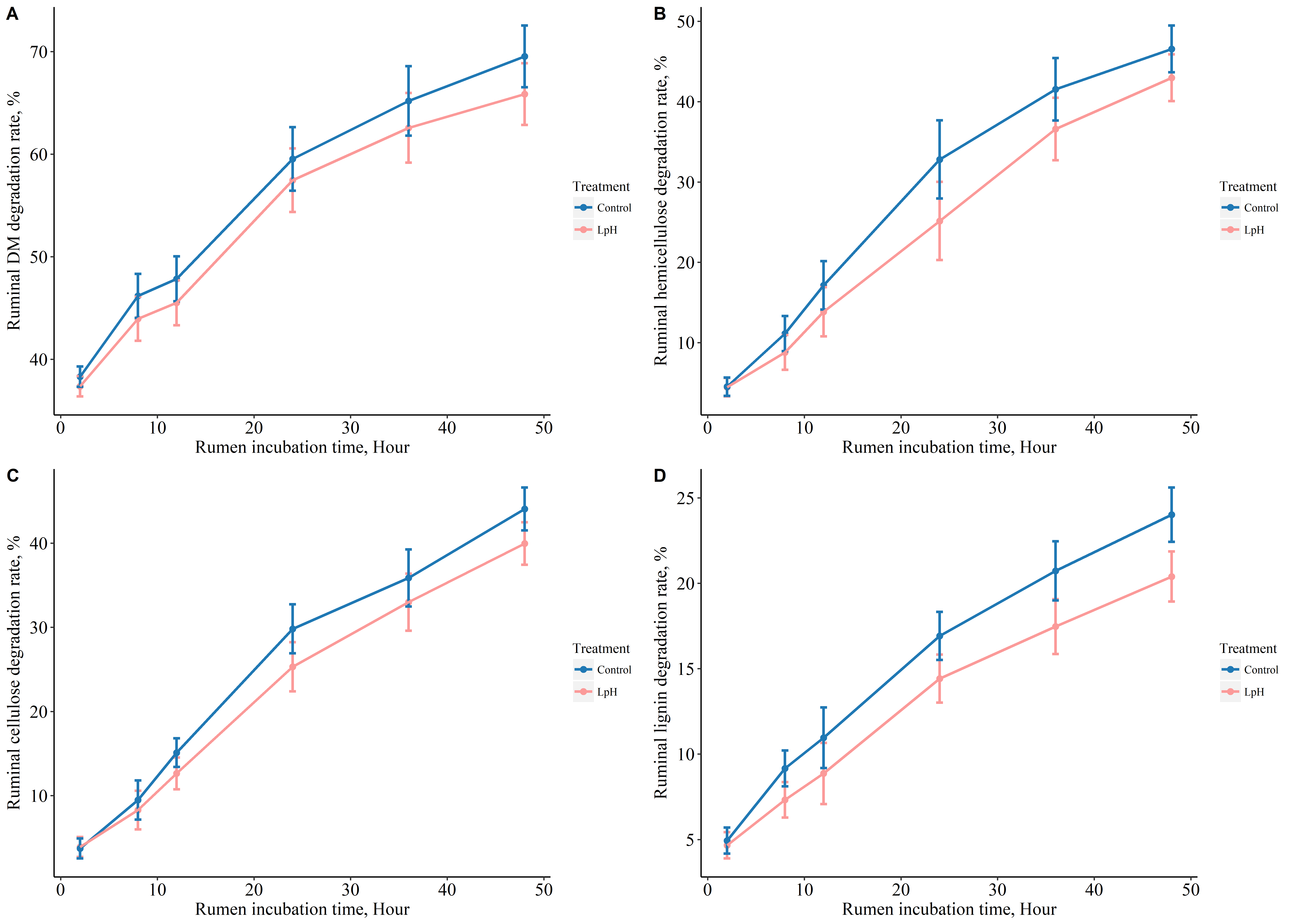


Figure S4. In situ degradation of dietary DM, hemicellulose, cellulose, and lignin with respect to the rumen incubation time.

Table S1. Ingredient composition and nutrient content of the ration^1^.

| Ingredient | % of DM |
| --- | --- |
| Corn silage | 33.54 |
| Chopped grass hay | 48.52 |
| Wheat middlings | 5.27 |
| Corn meal | 2.53 |
| Corn distillers dried grains | 1.01 |
| Corn gluten feed | 3.53 |
| Soybean meal | 3.33 |
| Soybean hulls | 1.67 |
| Limestone | 0.28 |
| Salt | 0.22 |
| Vitamin-mineral mix^2^ | 0.11 |
|  |  |
| Nutrient content | % of DM |
| DM (59.8% as fed) | 100 |
| CP | 12.6 |
| Ether extract | 5.30 |
| NDF | 46.2 |
| ADF | 23.6 |
| Hemicellulose | 22.6 |
| Cellulose | 21.0 |
| Lignin | 2.6 |
| Starch | 15.4 |
| Ash | 5.03 |

^1^Ingredients other than the corn silage and chopped hay were provided as a pelleted mix.

^2^Provided (per kg of pellet concentrate): vitamin A (9600 IU), vitamin D (3200 IU), vitamin E (41.2 mg), copper (26.9 mg), manganese (126 mg), zinc (200 mg), selenium (1.2 mg), monensin (27.2 mg), cobalt (0.1 mg), and iodine (2.1 mg).

Table S2. Effects of sampling site and ruminal pH on index of richness, alpha diversity, and evenness among treatments at bacterial and protozoal genera level.

| Variable | Control | | LpH | | SEM | *P* value | | |
| --- | --- | --- | --- | --- | --- | --- | --- | --- |
|  | Liquid | Solid | Liquid | Solid |  | Con vs LpH^1^ | Con vs LpH^2^ | Liq vs Sol^3^ |
| Bacterial genera |  |  |  |  |  |  |  |  |
| Observed species | 68 | 116 | 83 | 115 | 8 | 0.10 | 0.91 | < 0.001 |
| Richness index (Menhinick's) | 0.19 | 0.19 | 0.22 | 0.19 | 0.01 | 0.02 | 0.87 | 0.04 |
| Evenness index (Hill's ratio) | 0.04 | 0.02 | 0.03 | 0.02 | 0.003 | 0.09 | 0.97 | < 0.001 |
| Alpha diversity (Shannon-Wiener) | 2.64 | 3.11 | 2.85 | 3.07 | 0.09 | 0.04 | 0.74 | < 0.001 |
| Protozoal genera |  |  |  |  |  |  |  |  |
| Observed species | 9 | 9 | 10 | 9 | 1 | 0.15 | 0.83 | 0.23 |
| Richness index (Menhinick's) | 0.96 | 0.95 | 1.08 | 0.97 | 0.06 | 0.15 | 0.78 | 0.31 |
| Evenness index (Hill's ratio) | 0.15 | 0.19 | 0.13 | 0.19 | 0.02 | 0.28 | 0.83 | 0.004 |
| Alpha diversity (Shannon-Wiener) | 0.65 | 1.06 | 0.66 | 1.01 | 0.14 | 0.96 | 0.79 | 0.005 |

^1^Control versus LpH within the ruminal liquid fraction

^2^Control versus LpH within the ruminal solid fraction

^3^Ruminal sample fraction effect regardless of ruminal pH

Table S3. The effect of sampling site and treatment on bacterial phyla^1^.

| Variable | Control | | LpH | | SEM | *P* value | | |
| --- | --- | --- | --- | --- | --- | --- | --- | --- |
|  | Liquid | Solid | Liquid | Solid |  | Con vs LpH^2^ | Con vs LpH^3^ | Liq vs Sol^4^ |
| *Actinobacteria* | 0.13 | 0.78 | 0.30 | 0.65 | 0.12 | 0.32 | 0.46 | < 0.001 |
| *Bacteroidetes* | 4.60 | 3.60 | 3.89 | 3.46 | 0.15 | < 0.001 | 0.45 | < 0.001 |
| *Chloroflexi* | 0.10 | 0.45 | 0.33 | 0.39 | 0.08 | 0.05 | 0.58 | 0.02 |
| *Cyanobacteria* | 0.79 | 0.23 | 0.50 | 0.16 | 0.16 | 0.14 | 0.72 | 0.004 |
| *Firmicutes* | 4.21 | 4.74 | 3.94 | 4.38 | 0.13 | 0.17 | 0.07 | 0.002 |
| *Kiritimatiellaeota* | 2.86 | 1.27 | 2.69 | 1.47 | 0.31 | 0.71 | 0.64 | < 0.001 |
| *Lentisphaerae* | 2.89 | 1.92 | 2.81 | 1.99 | 0.26 | 0.83 | 0.86 | 0.003 |
| *Patescibacteria* | 0.23 | 0.22 | 0.12 | 0.30 | 0.03 | 0.04 | 0.19 | 0.04 |
| *Proteobacteria* | 5.21 | 4.47 | 3.93 | 4.22 | 0.23 | < 0.001 | 0.37 | 0.27 |
| *Spirochaetes* | 3.07 | 3.78 | 2.87 | 3.66 | 0.13 | 0.30 | 0.53 | < 0.001 |
| *Verrucomicrobia* | 1.55 | 0.67 | 1.23 | 0.61 | 0.34 | 0.43 | 0.88 | 0.02 |

^1^Data are centered log ratios of sequence counts of the bacterial phyla

^2^Control versus LpH within the ruminal liquid fraction

^3^Control versus LpH within the ruminal solid fraction

^4^Ruminal sample fraction effect regardless of ruminal pH

Table S4. Bacterial genera that were significantly different among treatments^1^.

| Variable | Control | | LpH | | SEM | *P* value | | |
| --- | --- | --- | --- | --- | --- | --- | --- | --- |
|  | Liquid | Solid | Liquid | Solid |  | Con vs LpH^2^ | Con vs LpH^3^ | Liq vs Sol^4^ |
| *Actinobacteria* |  |  |  |  |  |  |  |  |
| *Atopobium* | 0.62 | 1.17 | 0.76 | 1.79 | 0.40 | 0.77 | 0.20 | 0.03 |
| *Bifidobacterium* | 0.20 | 0.57 | 1.18 | 1.82 | 0.43 | 0.11 | 0.04 | 0.22 |
| *Olsenella* | 1.22 | 3.25 | 1.42 | 2.90 | 0.34 | 0.68 | 0.47 | < 0.001 |
| *Bacteroidetes* |  |  |  |  |  |  |  |  |
| *Prevotella* | 0.82 | 0.59 | 0.12 | 0.03 | 0.24 | 0.04 | 0.10 | 0.48 |
| *Prevotella_1* | 6.78 | 5.66 | 6.05 | 5.60 | 0.27 | 0.07 | 0.88 | 0.01 |
| *Prevotella_9* | 2.70 | 1.83 | 1.12 | 3.02 | 0.55 | 0.02 | 0.07 | 0.25 |
| *Prevotellaceae_NK3B31_group* | 0.48 | 1.47 | 0.67 | 1.71 | 0.40 | 0.75 | 0.67 | 0.02 |
| *Prevotellaceae_UCG-001* | 2.16 | 3.18 | 2.17 | 3.59 | 0.38 | 0.97 | 0.31 | < 0.001 |
| *Prevotellaceae_UCG-004* | 0.19 | 0.72 | 0.37 | 0.71 | 0.21 | 0.54 | 0.98 | 0.04 |
| *Prevotellaceae_YAB2003_group* | 2.59 | 0.23 | 2.40 | 1.34 | 0.49 | 0.79 | 0.13 | < 0.001 |
| *Chloroflexi* |  |  |  |  |  |  |  |  |
| *Flexilinea* | 1.34 | 2.96 | 2.84 | 2.89 | 0.32 | 0.01 | 0.89 | 0.02 |
| *Elusimicrobia* |  |  |  |  |  |  |  |  |
| *Elusimicrobium* | 3.09 | 1.65 | 3.47 | 1.85 | 0.47 | 0.53 | 0.73 | < 0.001 |
| *Firmicutes* |  |  |  |  |  |  |  |  |
| *Acetitomaculum* | 2.04 | 4.19 | 2.68 | 3.50 | 0.51 | 0.39 | 0.35 | 0.01 |
| *Anaerosporobacter* | 4.40 | 4.23 | 2.67 | 3.87 | 0.48 | 0.01 | 0.55 | 0.22 |
| *Anaerovibrio* | 0.22 | 0.05 | 0.19 | 0.06 | 0.03 | 0.44 | 0.75 | < 0.001 |
| *Asteroleplasma* | 3.40 | 1.55 | 3.29 | 2.63 | 0.50 | 0.88 | 0.14 | 0.02 |
| *Butyrivibrio_2* | 1.44 | 3.61 | 1.86 | 3.54 | 0.42 | 0.49 | 0.91 | < 0.001 |
| *Christensenellaceae_R-7_group* | 5.60 | 6.27 | 6.00 | 6.01 | 0.16 | 0.09 | 0.26 | 0.04 |
| *Erysipelotrichaceae_UCG-004* | 6.09 | 5.27 | 6.45 | 5.65 | 0.24 | 0.30 | 0.27 | < 0.001 |
| *Family_XIII_AD3011_group* | 0.32 | 2.46 | 0.70 | 1.90 | 0.29 | 0.36 | 0.19 | < 0.001 |
| *Family_XIII_UCG-001* | 0.33 | 1.74 | 0.52 | 1.45 | 0.27 | 0.62 | 0.45 | < 0.001 |
| *Lachnoclostridium_1* | 0.15 | 1.47 | 0.14 | 0.36 | 0.18 | 0.98 | < 0.001 | < 0.001 |
| *Lachnoclostridium_10* | 0.16 | 1.19 | 0.58 | 0.94 | 0.32 | 0.36 | 0.59 | 0.04 |
| *Lachnospiraceae_AC2044_group* | 0.21 | 2.28 | 0.83 | 2.10 | 0.27 | 0.11 | 0.63 | < 0.001 |
| *Lachnospiraceae_NK3A20_group* | 2.33 | 5.45 | 2.76 | 4.79 | 0.67 | 0.66 | 0.49 | < 0.001 |
| *Lachnospiraceae_NK4A136_group* | 0.19 | 0.75 | 0.18 | 0.94 | 0.15 | 0.94 | 0.37 | < 0.001 |
| *Lachnospiraceae_UCG-007* | 1.12 | 1.07 | 0.15 | 1.12 | 0.33 | 0.05 | 0.91 | 0.17 |
| *Lachnospiraceae_XPB1014_group* | 0.19 | 2.11 | 0.49 | 1.21 | 0.32 | 0.51 | 0.06 | < 0.001 |
| *Mogibacterium* | 0.65 | 3.84 | 1.94 | 3.40 | 0.34 | 0.01 | 0.37 | < 0.001 |
| *Moryella* | 0.46 | 3.82 | 1.15 | 3.05 | 0.39 | 0.23 | 0.18 | < 0.001 |
| *Papillibacter* | 0.74 | 2.18 | 2.50 | 2.47 | 0.45 | 0.01 | 0.65 | 0.14 |
| *Pseudobutyrivibrio* | 1.26 | 2.04 | 1.04 | 2.83 | 0.38 | 0.70 | 0.16 | < 0.001 |
| *Ruminiclostridium_9* | 0.19 | 1.28 | 0.15 | 0.61 | 0.24 | 0.90 | 0.05 | < 0.001 |
| *Ruminococcaceae_UCG-004* | 0.36 | 1.77 | 0.41 | 1.37 | 0.34 | 0.93 | 0.42 | < 0.001 |
| *Ruminococcaceae_UCG-010* | 2.52 | 2.29 | 4.07 | 2.67 | 0.43 | 0.02 | 0.55 | 0.07 |
| *Ruminococcaceae_UCG-014* | 1.39 | 2.64 | 1.30 | 2.19 | 0.54 | 0.89 | 0.51 | 0.04 |
| *Ruminococcus_1* | 4.35 | 5.96 | 4.04 | 5.60 | 0.30 | 0.48 | 0.41 | < 0.001 |
| *Ruminococcus_2* | 5.27 | 6.04 | 5.80 | 5.99 | 0.25 | 0.10 | 0.86 | 0.04 |
| *Saccharofermentans* | 3.25 | 4.62 | 3.24 | 4.46 | 0.27 | 0.99 | 0.70 | < 0.001 |
| *Selenomonas_1* | 0.74 | 1.99 | 1.23 | 2.45 | 0.35 | 0.30 | 0.33 | < 0.001 |
| *Lentisphaerae* |  |  |  |  |  |  |  |  |
| *Victivallis* | 0.19 | 0.04 | 1.09 | 0.35 | 0.27 | 0.03 | 0.44 | 0.12 |
| *Proteobacteria* |  |  |  |  |  |  |  |  |
| *Bibersteinia* | 0.17 | 0.04 | 0.31 | 0.11 | 0.07 | 0.13 | 0.38 | 0.02 |
| *Desulfovibrio* | 1.09 | 2.74 | 1.67 | 2.62 | 0.34 | 0.15 | 0.75 | < 0.001 |
| *Desulfuromonas* | 0.21 | 0.35 | 0.17 | 0.06 | 0.10 | 0.73 | 0.03 | 0.87 |
| *Pseudomonas* | 1.25 | 0.15 | 0.15 | 0.62 | 0.26 | 0.01 | 0.21 | 0.24 |
| *Ruminobacter* | 6.53 | 5.86 | 6.15 | 5.76 | 0.30 | 0.26 | 0.75 | 0.03 |
| *Succinimonas* | 6.26 | 5.74 | 4.44 | 5.35 | 0.53 | 0.02 | 0.56 | 0.69 |
| *Succinivibrio* | 4.46 | 3.25 | 4.31 | 3.74 | 0.37 | 0.77 | 0.37 | 0.03 |
| *Succinivibrionaceae_UCG-002* | 8.29 | 7.44 | 6.05 | 7.23 | 0.52 | < 0.001 | 0.75 | 0.72 |
| *Spirochaetes* |  |  |  |  |  |  |  |  |
| *M2PT2-76_termite_group* | 1.45 | 1.80 | 0.72 | 0.07 | 0.48 | 0.06 | < 0.001 | 0.55 |
| *Sediminispirochaeta* | 2.68 | 3.10 | 3.65 | 3.05 | 0.28 | 0.03 | 0.89 | 0.76 |
| *Sphaerochaeta* | 3.89 | 3.28 | 3.91 | 3.35 | 0.28 | 0.97 | 0.85 | 0.05 |
| *Treponema* | 2.69 | 3.65 | 3.64 | 3.85 | 0.33 | 0.04 | 0.64 | 0.07 |
| *Treponema_2* | 6.36 | 7.20 | 6.60 | 7.30 | 0.20 | 0.42 | 0.73 | < 0.001 |
| *Synergistetes* |  |  |  |  |  |  |  |  |
| *Pyramidobacter* | 0.71 | 2.28 | 1.64 | 2.18 | 0.31 | 0.03 | 0.80 | < 0.001 |
| *Synergistes* | 0.20 | 0.17 | 0.62 | 0.06 | 0.12 | 0.02 | 0.50 | 0.03 |
| *Tenericutes* |  |  |  |  |  |  |  |  |
| *Mycoplasma* | 0.19 | 0.04 | 0.15 | 0.07 | 0.03 | 0.25 | 0.47 | < 0.001 |
| *Verrucomicrobia* |  |  |  |  |  |  |  |  |
| *Cerasicoccus* | 0.60 | 0.14 | 1.00 | 0.36 | 0.26 | 0.29 | 0.55 | 0.05 |

^1^Sequence counts of the bacterial genera were transformed to centered log ratio to avoid compositional data problem

^2^Control versus LpH within the ruminal liquid fraction

^3^Control versus LpH within the ruminal solid fraction

^4^Ruminal sample fraction effect regardless of ruminal pH

Table S5. Protozoal genera that differed among treatments^1^

| Variable | Control | | LpH | | SEM | *P* value | | |
| --- | --- | --- | --- | --- | --- | --- | --- | --- |
|  | Liquid | Solid | Liquid | Solid |  | Con vs LpH^2^ | Con vs LpH^3^ | Liq vs Sol^4^ |
| *Diploplastron* | 0.16 | 0.20 | 0.13 | 0.28 | 0.05 | 0.63 | 0.27 | 0.04 |
| *Entodinium* | 6.60 | 5.81 | 5.70 | 5.77 | 0.36 | 0.02 | 0.92 | 0.20 |
| *Eudiplodinium* | 2.09 | 3.06 | 1.85 | 3.04 | 0.29 | 0.51 | 0.95 | 0.001 |
| *Isotricha* | 4.09 | 3.90 | 2.22 | 3.65 | 0.46 | 0.004 | 0.70 | 0.18 |

^1^Sequence counts of the protozoal genera were transformed to centered log ratio to avoid compositional data problem

^2^Control versus LpH within the ruminal liquid fraction

^3^Control versus LpH within the ruminal solid fraction

^4^Ruminal sample fraction effect regardless of ruminal pH

Table S6. Archaea genera that differed among treatments^1^

| Variable | Control | | LpH | | SEM | *P* value | | |
| --- | --- | --- | --- | --- | --- | --- | --- | --- |
|  | Liquid | Solid | Liquid | Solid |  | Con vs LpH^2^ | Con vs LpH^3^ | Liq vs Sol^4^ |
| *Candidatus_Methanomethylophilus* | 2.72 | 2.28 | 2.69 | 2.25 | 0.22 | 0.91 | 0.93 | 0.05 |
| *Methanobrevibacter* | 1.39 | 2.13 | 1.56 | 2.02 | 0.19 | 0.38 | 0.56 | < 0.001 |
| *Methanosphaera* | 0.03 | 0.02 | 0.02 | 0.03 | 0.01 | 0.71 | 0.83 | 0.72 |

^1^Sequence counts of the bacterial genera were transformed to centered log ratio to avoid compositional data problem.

^2^Control versus LpH within the ruminal liquid fraction

^3^Control versus LpH within the ruminal solid fraction

^4^Ruminal sample fraction effect regardless of ruminal pH

Table S7. Carbohydrate-active degrading enzymes that differed among treatments^1^.

| Variable | Control | | LpH | | SEM | *P* value | | |
| --- | --- | --- | --- | --- | --- | --- | --- | --- |
|  | Liquid | Solid | Liquid | Solid |  | Con vs LpH^3^ | Con vs LpH^4^ | Liq vs Sol^5^ |
| Alpha-glucosidase (EC 3.2.1.20)^2^ | 3.31 | 2.13 | 2.99 | 2.04 | 0.37 | 0.53 | 0.85 | 0.003 |
| Amylase (EC 3.2.1.1) | 5.31 | 3.75 | 5.03 | 3.39 | 0.47 | 0.65 | 0.56 | < 0.001 |
| Beta-galactosidase (EC 3.2.1.23) | 2.01 | 1.79 | 2.67 | 1.46 | 0.37 | 0.16 | 0.49 | 0.03 |
| Cellulase celA (EC 3.2.1.4) | 2.73 | 1.37 | 2.77 | 1.31 | 0.52 | 0.95 | 0.91 | 0.001 |
| Glucan 1,4-alpha-maltotetraohydrolase (EC 3.2.1.60) | 0.02 | 0.90 | 0.001 | 0.64 | 0.26 | 0.97 | 0.47 | 0.003 |
| Glucan phosphorylase (ACM90985.1) | 0.83 | 2.14 | 1.69 | 1.91 | 0.35 | 0.05 | 0.61 | 0.01 |
| Glucosidase Cel1C (AAP30745.1) | 0.91 | 0.63 | 2.21 | 1.09 | 0.40 | 0.01 | 0.38 | 0.06 |
| Glycoside hydrolase family 43 (ACX75355.1) | 0.30 | 3.17 | 1.44 | 3.07 | 0.53 | 0.11 | 0.89 | < 0.001 |
| Glycosyl hydrolase family 16 (ADE81965.1) | 1.16 | 0.84 | 0.13 | 0.41 | 0.31 | 0.02 | 0.31 | 0.95 |
| Glycosyl hydrolase family 43 (ADE82026.1) | 2.00 | 3.51 | 2.60 | 1.81 | 0.53 | 0.42 | 0.02 | 0.49 |
| Glycosyl hydrolase family 51 (ADE81862.1) | 0.43 | 1.28 | 0.72 | 1.00 | 0.26 | 0.37 | 0.38 | 0.01 |
| Glycosyl hydrolase family 57 (ADE82175.1) | 3.08 | 2.13 | 2.67 | 2.30 | 0.27 | 0.27 | 0.64 | 0.01 |
| Glycosyltransferase 36 (ADU20744.1) | 0.52 | 2.32 | 0.89 | 1.58 | 0.37 | 0.48 | 0.16 | 0.001 |
| Isoamylase domain/esterase family protein (ADE82534.1) | 0.29 | 1.09 | 0.53 | 0.57 | 0.20 | 0.40 | 0.07 | 0.03 |
| Pectate lyase (EC 4.2.2.2) | 0.16 | 0.53 | 1.11 | 1.22 | 0.23 | < 0.001 | < 0.001 | 0.16 |
| Penicillin-binding protein 1A (ADE81110.1) | 1.77 | 0.99 | 2.13 | 1.38 | 0.38 | 0.51 | 0.47 | 0.04 |
| Putative 4-alpha-glucanotransferase (ADE83753.1) | 3.96 | 3.52 | 3.82 | 2.91 | 0.25 | 0.68 | 0.07 | 0.005 |
| Putative alpha-xylosidase (ADE82775.1) | 3.00 | 2.47 | 2.90 | 2.08 | 0.31 | 0.82 | 0.36 | 0.03 |
| Putative carbohydrate-active enzyme (ADD61402.1) | 2.99 | 1.90 | 3.24 | 1.72 | 0.42 | 0.59 | 0.71 | < 0.001 |
| Putative glycosyl transferase (ADE81144.1) | 2.16 | 1.03 | 0.75 | 1.65 | 0.46 | 0.03 | 0.33 | 0.80 |
| Rhamnogalacturonan acetylesterase (CAA61858.1) | 0.05 | 0.20 | 0.70 | 0.24 | 0.26 | 0.05 | 0.90 | 0.51 |
| Sucrose alpha-glucosidase (EC 3.2.1.48) | 2.36 | 0.32 | 0.13 | 0.24 | 0.41 | < 0.001 | 0.88 | 0.01 |
| UDP-3-0-acyl N-acetylglucosamine deacetylase (ADE83477.1) | 1.46 | 2.00 | 2.46 | 1.44 | 0.34 | 0.04 | 0.24 | 0.47 |

^1^Sequence counts of the enzyme expression were transformed to centered log ratio to avoid compositional data problem.

^2^accession number

^3^Control versus LpH within the ruminal liquid fraction

^4^Control versus LpH within the ruminal solid fraction

^5^Ruminal sample fraction effect regardless of ruminal pH

Table S8. Effects of ruminal pH on DMI and ruminal short chain fatty acid concentrations.

| Variable | Treatment | | SEM | P value |
| --- | --- | --- | --- | --- |
|  | Control | LpH |  |  |
| pH | 6.44 | 6.09 | 0.04 | < 0.0001 |
| DMI, kg/d | 9.75 | 7.82 | 0.66 | 0.04 |
| Total VFA, mM | 82.84 | 62.31 | 3.80 | 0.001 |
| Acetate, mM | 54.71 | 42.12 | 2.38 | 0.001 |
| Propionate, mM | 20.05 | 14.61 | 1.24 | 0.001 |
| Butyrate, mM | 5.94 | 3.96 | 0.50 | 0.001 |
| Isobutyrate, Mm | 0.79 | 0.58 | 0.05 | 0.001 |
| Valerate, mM | 0.68 | 0.51 | 0.06 | 0.04 |
| Isovalerate, Mm | 0.43 | 0.31 | 0.03 | 0.003 |

Table S9. Effects of ruminal pH change on in situ fiber degradation kinetics^1^.

| Variable | Treatment | | SEM | *P* value |
| --- | --- | --- | --- | --- |
|  | Control | LpH |  |  |
| DM |  |  |  |  |
| a | 35.15 | 34.14 | 0.94 | 0.31 |
| b | 47.19 | 45.84 | 6.11 | 0.81 |
| K_d_ | 3.30 | 3.60 | 1.20 | 0.78 |
| ED | 50.62 | 48.76 | 0.64 | 0.06 |
| Hemicellulose |  |  |  |  |
| a | -0.79 | -0.03 | 0.78 | 0.43 |
| b | 65.97 | 79.19 | 7.80 | 0.13 |
| K_d_ | 3.30 | 2.00 | 0.50 | 0.09 |
| ED | 20.53 | 17.30 | 0.74 | 0.02 |
| Cellulose |  |  |  |  |
| a | -0.08 | 0.50 | 0.94 | 0.33 |
| b | 69.78 | 72.81 | 5.61 | 0.61 |
| K_d_ | 2.30 | 1.80 | 0.20 | 0.15 |
| ED | 18.15 | 16.24 | 0.63 | 0.02 |
| Lignin |  |  |  |  |
| a | 3.49 | 3.19 | 0.34 | 0.49 |
| b | 33.03 | 35.77 | 8.39 | 0.75 |
| K_d_ | 2.70 | 2.20 | 0.50 | 0.50 |
| ED | 12.12 | 10.29 | 0.31 | 0.002 |

^1^a represents proportion of rapidly degradable fraction (%); b represents proportion of potentially degradable fraction (%); K_d_ represents the degradation rate constant of b (%/h); ED is effective degradability (%).
